# Estimation with Heisenberg-Scaling Sensitivity of a Single Parameter Distributed in an Arbitrary Linear Optical Network

**DOI:** 10.3390/s22072657

**Published:** 2022-03-30

**Authors:** Danilo Triggiani, Vincenzo Tamma

**Affiliations:** 1 School of Mathematics and Physics, University of Portsmouth, Portsmouth PO1 3QL, UK; danilo.triggiani@port.ac.uk; 2 Institute of Cosmology and Gravitation, University of Portsmouth, Portsmouth PO1 3FX, UK

**Keywords:** quantum metrology, quantum sensing, distributed parameter, heisenberg limit, gaussian metrology, squeezing

## Abstract

Quantum sensing and quantum metrology propose schemes for the estimation of physical properties, such as lengths, time intervals, and temperatures, achieving enhanced levels of precision beyond the possibilities of classical strategies. However, such an enhanced sensitivity usually comes at a price: the use of probes in highly fragile states, the need to adaptively optimise the estimation schemes to the value of the unknown property we want to estimate, and the limited working range, are some examples of challenges which prevent quantum sensing protocols to be practical for applications. This work reviews two feasible estimation schemes which address these challenges, employing easily realisable resources, i.e., squeezed light, and achieve the desired quantum enhancement of the precision, namely the Heisenberg-scaling sensitivity. In more detail, it is here shown how to overcome, in the estimation of any parameter affecting in a distributed manner multiple components of an arbitrary *M*-channel linear optical network, the need to iteratively optimise the network. In particular, we show that this is possible with a single-step adaptation of the network based only on a prior knowledge of the parameter achievable through a “classical” shot-noise limited estimation strategy. Furthermore, homodyne measurements with only one detector allow us to achieve Heisenberg-limited estimation of the parameter. We further demonstrate that one can avoid the use of any auxiliary network at the price of simultaneously employing multiple detectors.

## 1. Introduction

Due to the discrete nature of physical phenomena, the error in the estimation of physical properties, such as lengths, delays, temperatures, or refractive indexes, when employing *N* probes (e.g., photons, electrons) is strongly limited by the so-called shot-noise scaling factor of $1/\sqrt{N}$ when a classical estimation strategy, i.e., in which the probe and the measurement employed can be fully described classically, is performed. However, it has been proven that it is possible, by exploiting quantum features such as entanglement and the squeezing of light, to overcome this classical limitation, and to reach an enhanced scaling in the precision of order 1/N, called the Heisenberg limit [1,2,3,4,5,6,7,8,9,10,11,12,13,14,15,16,17].

A promising path towards this quantum-enhanced sensitivity is the one enabled by the squeezing of light [18,19,20,21]. Squeezed states are particular states of the electromagnetic field characterised by a quadrature field with smaller fluctuations than the field quadratures of the vacuum itself. This useful property, together with the Gaussian nature of these states which makes them relatively easy to produce, resilient to noise, and mathematically simple to manipulate [20,22], makes these states evident candidates for metrological purposes. The working principle of these protocols is rather straightforward, and can be outlined as follows. The probe, a pure squeezed state, undergoes an optical phase delay of a magnitude which depends on the unknown parameter we are interested to measure. The phase delay causes a transformation, or more precisely a rotation, of the state of the probe, that needs to be observed to infer the unknown parameter. In order to obtain the quantum enhancement, the measured quadrature field must be ‘sufficiently’ squeezed, namely it must possess a variance reduced below the vacuum fluctuations.

It is possible to highlight two major different approaches undertaken in literature, according to what type of squeezed state is employed and, ultimately, to how the information about the parameter is encoded on, and then retrieved from, the probe. In ‘displacement-encoding’ approaches [2,23], the initial overall state possesses a non-vanishing displacement—i.e., the average of the quadrature fields—so that the value of the parameter is encoded into the variation of the displacement. This approach presents the advantage that the unknown parameter can be retrieved through the measurement of the displacement of the probe, namely the mean value of the signal experimentally observed in a laboratory when performing homodyne detection. In ‘squeezing-encoding’ approaches [24,25,26], all the resources are accumulated in the squeezing of the probes, which are thus squeezed vacuum states, so that the information on the parameter is encoded into the parameter-dependent rotation of the covariance matrix. This is the optimal approach for squeezing-based estimation protocols in the sense that, for a fixed total average number of photons in a Gaussian state, the strategy that maximises the precision is to concentrate all the photons in the squeezing [24,26,27]. Since the information about the parameter is encoded in the covariance matrix of the probe, with this approach the estimation consists of retrieving the value of the parameter from the modulation of the noise. Another interesting approach involves the use of an active component for the estimation of phases, i.e., anti-squeezing the signal before the detection [28,29,30,31,32]. In this approach, the goal is generally to estimate the value of the unknown phase detecting whether the final state of the probe is different from the one injected in the network, namely, performing a projective measurement on the initial state.

Another interesting aspect of quantum metrology that has been frequently investigated is the possibility to estimate a parameter which is not localised into a single node of the network which encodes it, but distributed in an arbitrary manner among multiple components. Although this framework entails a further complication, given by the generally non-trivial encoding of the value of the parameter onto the probe, it allows for the study of estimation schemes with arbitrary structures of the networks. Since, in this approach, the network is generally described as a ‘black-box’, namely the structure of the network is not specified, the applicability of the estimation technique is universal—i.e., guaranteed for any passive and unitary evolution of the probe—covering in this way experimental situations rarely treated in literature. Moreover, the absence of a defined network structure allows to analyse general properties, such as the average performance of the estimation over some set of local transformations [33], the best possible Gaussian strategy for a multi-channel network [27], or the typicality of the Heisenberg scaling [34]. Applications range from high-precision biomedical phase imaging, quantum enhanced pattern recognition, and gravitational-waves sensing, to the mapping of external fields (magnetic and electric fields or temperatures).

However, standard Gaussian estimation protocols still present some challenges. In particular, adaptivity—i.e., the fact that a scheme for the estimation of the parameter depends on the parameter itself—is known to be a typical feature of ab initio Gaussian metrology [24,35,36,37], namely schemes for the estimation of a parameter of which no prior information is known. Intuitively, the cause of the adaptivity stems from the fact that the phase of the squeezed-quadrature which needs to be measured to achieve the quantum-enhanced precision depends on the phase acquired by the probe through the interferometric evolution in arbitrary parameter-encoding networks and, ultimately, on the unknown parameter. Moreover, in distributed quantum metrology, a further experimental challenge arises from the need to adaptively optimize the preparation of the probe and of the measurement [27]. A common approach to avoid adaptivity is limiting the range of values that the unknown parameter is allowed to take, for example, requiring that the parameter is small [38,39,40,41]. In fact, in the regime of small phases, the transformation the probe undergoes is small enough to make the rotation of the squeezed quadrature negligible, which in turn remains practically unchanged. Nevertheless, in some practical scenarios the experimenters have no control on the value of the phase—e.g., the system under investigation is unreachable or cannot be manipulated—and in such cases, this approach ceases to be feasible.

**Figure 1:**
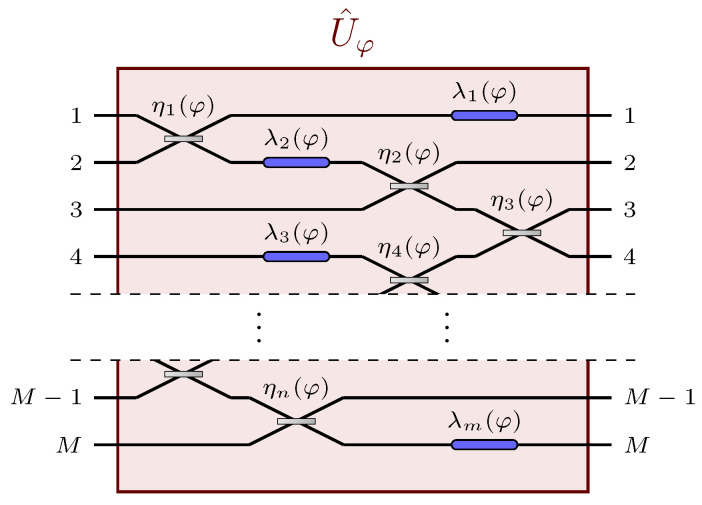
Example of a passive and linear network ${\hat{U}}_{\varphi }$ which depends on a single global parameter φ. The parameter can be thought of as a physical property of an external agent (e.g., temperature, electromagnetic field) which affects multiple components, possibly of different natures, of the network [42,43]. Reprinted with permission from ref. [42], © 2021 The Author(s).

We will show here how the challenge of adaptivity, and any restriction in the range of possible values of the parameter to be estimated, can be overcome thanks to some recent results in the field of distributed quantum metrology, for the estimation at the Heisenberg scaling sensitivity of a single parameter, distributed throughout an arbitrary, multi-channel passive and linear network (see Figure 1). In particular, the unknown parameter can be thought of as a physical property of an external field—such as the temperature of the environment, or the magnitude of an electromagnetic field—which globally affects some or all of the components of the network. This review is organised in two parts, in which we introduce two distinct estimation schemes, discussing the features that differentiate the two approaches. In Section 2, we will discuss a squeezing-encoding scheme achieving Heisenberg-scaling sensitivity by employing a single squeezed vacuum state and homodyne detection at a single output channel [42]. We will assume that no prior knowledge on the value of the parameter is known, and that the structure of the network, as well as the nature of the parameter it encodes, are completely arbitrary. We will present the conditions that need to be satisfied in order to reach the Heisenberg scaling in such a generic model, and we will show that, in general, only a classical prior estimation of the unknown parameter suffices to prepare a single auxiliary linear network required to optimize the setup. These results show that it is possible to conceive feasible two-step estimation strategies, composed of a first classical estimation of the unknown parameter required to engineer the auxiliary stage, and then the actual quantum estimation. In Section 3, we describe a different scheme achieving the Heisenberg scaling, which makes use of a single-mode squeezed-coherent state as a probe, and homodyne detection in every output port of the linear network [43]. Once again, the model will assume no prior knowledge on the parameter, nor on the structure of the network. We will see that, in this case, no auxiliary stage is required to achieve the Heisenberg scaling, so that the estimation can be carried out in a single step, at the cost of employing a multiple homodyne detector. Moreover, we will show that, due to the introduction of a non-vanishing displacement in the probe, the overall precision becomes the sum of two contributions, one deriving from the information encoded in the sample mean of the outcomes of the homodyne measurements, the other in the sample covariance matrix.

## 2. Quantum Estimation Based on Single-Homodyne Measurements

In this section, we will describe a generic model of a *M*-channel linear network ${\hat{U}}_{\varphi }$ that allows the Heisenberg scaling to be reached in the estimation of a parameter φ distributed arbitrarily in ${\hat{U}}_{\varphi }$, regardless of the structure of the network. Our model makes use of two auxiliary stages, namely two other linear networks ${\hat{V}}_{\mathrm{in}}$ and ${\hat{V}}_{\mathrm{out}}$, whose purposes are to distribute the probe, a single-mode squeezed vacuum injected in the first channel, in all the optical modes of ${\hat{U}}_{\varphi }$, and then to refocus it in the only channel observed through homodyne detection. We will show that only one of the two auxiliary networks must be optimized, and although the optimal choice of such network depends on the value of φ, the required precision for this optimization can be achieved with a classical estimation strategy. This allows us to conceive two-step estimation protocols, where a prior classical estimation to engineer the optimal auxiliary stage is followed by the quantum Heisenberg scaling-achieving estimation.

### 2.1. Setup

Let us consider a *M*-channel passive linear network, whose action on the state of the probe is described by the unitary operator ${\hat{U}}_{\varphi }$, φ being a single, generally distributed, unknown parameter we are interested to estimate. Due to its passive and linear nature, this network can be represented by a unitary matrix. Let then, $U_{\varphi }$ be the M×M unitary matrix representing the action of ${\hat{U}}_{\varphi }$ on the annihilation operators ${\hat{a}}_{i}$, i=1,…,M, associated with each channel of the network, satisfying the commutation relations $[{\hat{a}}_{i}{\hat{a}}_{j}]=0$, $[{\hat{a}}_{i}{\hat{a}}_{j}^{†}]=\delta_{ij}$, where we denote with $\delta_{ij}$ the Kronecker delta. The matrix $U_{\varphi }$ is thus defined by the transformation

(1) $${\hat{U}}_{\varphi }^{†}{\hat{a}}_{i}^{†}{\hat{U}}_{\varphi }=\sum_{j}{\left(U_{\varphi }\right)}_{ij}{\hat{a}}_{j}^{†}.$$

We will consider a single-mode squeezed state

(2) $$|\psi_{0}\rangle =|r\rangle \equiv \hat{S}\left(r\right)|\mathrm{vac}\rangle ,\;r=\left(r,0,\ldots ,0\right),$$

as a probe, with $\hat{S}\left(r\right)=e^{\frac{1}{2}r\left({\hat{a}}_{1}^{†2}-{\hat{a}}_{1}^{2}\right)}$ squeezing operator and $N=\sinh^{2}r$ average number of photons, all injected in a single channel of the apparatus, i.e., the first one with the choice of squeezing parameters r in Equation (2). In other words, the state $|\psi_{0}\rangle$ presents a non-vanishing number of photons only in the first mode. As discussed in Section 1, the approach of squeezing-based estimation strategies is to infer the value of φ from the transformation of the covariance matrix $\Gamma =\mathrm{diag}(e^{2r},1,\ldots ,e^{-2r},1,\ldots )/2$ of the state |ψ〉 after the interferometric evolution ${\hat{U}}_{\varphi }$. To do so, we will consider the model where a single output channel, say the first, is measured through homodyne detection. We will denote with θ the phase of the local oscillator, which coincides with the phase of the measured quadrature ${\hat{x}}_{\theta }$. We will assume that r>0 without loss of generality. We notice that, with this assumption, the squeezed quadrature of the state $|\psi_{0}\rangle$ is ${\hat{p}}_{1}\equiv -i\left({\hat{a}}_{1}-{\hat{a}}_{1}^{†}\right)/\sqrt{2}$, with $\mathrm{Var}\left[{\hat{p}}_{1}\right]=e^{-2r}/2$, while the anti-squeezed quadrature field is ${\hat{x}}_{1}\equiv \left({\hat{a}}_{1}+{\hat{a}}_{1}^{†}\right)/\sqrt{2}$, with $\mathrm{Var}\left[{\hat{x}}_{1}\right]=e^{2r}/2$. In terms of the creation and annihilation operators, the measured quadrature field can be expressed as ${\hat{x}}_{\theta }=\left(e^{-i\theta }{\hat{a}}_{1}+e^{i\theta }{\hat{a}}_{1}^{†}\right)/\sqrt{2}$.

**Figure 2:**
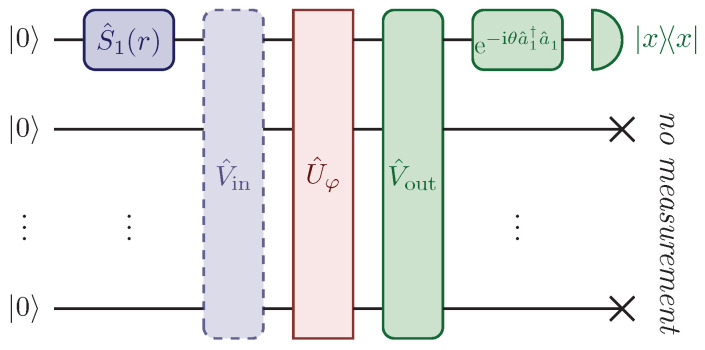
Schematic diagram of the setup described in Section 2.1. The squeezed vacuum state in Equation (2) is injected in the first channel of a network composed of a first auxiliary stage ${\hat{V}}_{\mathrm{in}}$, a network ${\hat{U}}_{\varphi }$ which depends on a generally distributed parameter φ we want to estimate, and a second auxiliary stage ${\hat{V}}_{\mathrm{out}}$, before being detected through homodyne measurements in the first output port. The role of the two auxiliary stages ${\hat{V}}_{\mathrm{in}}$ and ${\hat{V}}_{\mathrm{out}}$ is to respectively distribute the photons of the probe through multiple channels, and then to refocus them into the only observed channel. We will show that only one auxiliary network needs to be optimized to reach the Heisenberg scaling, while, for networks with a large number of channels, the effect of the non-optimized network is typically irrelevant on the overall precision of the estimation [42]. Reprinted with permission from ref. [42], © 2021 The Author(s).

Since the linear network ${\hat{U}}_{\varphi }$ is arbitrary, the average number of photons that can be actually detected after the interferometric evolution ranges between 0 and *N*. Naturally, if this number were to be small, or far from *N*, we would expect a sub-optimal performance of the estimation scheme, since most of the photons would come out of the network ${\hat{U}}_{\varphi }$ from channels that are not observed, and information on φ would in this way be lost. Moreover, it may happen that the transformation that it imposes on the probe in the transition to the first output port is trivial, namely that the element ${\left(U_{\varphi }\right)}_{11}$ of the transition amplitude matrix $U_{\varphi }$ does not depend on φ. This occurrence would preclude the probe from acquiring any observable information on the parameter. In order to prevent these conditions from happening, this model includes the presence of two auxiliary linear and passive networks acting on the probe, ${\hat{V}}_{\mathrm{in}}$ before and ${\hat{V}}_{\mathrm{out}}$ after the linear network ${\hat{U}}_{\varphi }$ (see Figure 2). The first auxiliary stage ${\hat{V}}_{\mathrm{in}}$ can be understood as network scattering, which distributes the probe through multiple input channels of the parameter-dependent network ${\hat{U}}_{\varphi }$. The purpose of the second stage ${\hat{V}}_{\mathrm{out}}$ is instead to refocus the probe, after the interaction with ${\hat{U}}_{\varphi }$, into the only observed channel. The unitary matrix describing the overall network is thus

(3) $$u_{\varphi }=V_{\mathrm{out}}U_{\varphi }V_{\mathrm{in}},$$

given by the matrix product of the three single matrix representations $V_{\mathrm{out}}$, $U_{\varphi }$ and $V_{\mathrm{in}}$.

Since in this model all the photons are injected in the first input channel, which is also the only channel observed at the output, the only relevant transition amplitude is the element ${\left(u_{\varphi }\right)}_{11}$ of the overall unitary matrix in Equation (3). We can then rewrite

(4) $${\left(u_{\varphi }\right)}_{11}={\left(V_{\mathrm{out}}U_{\varphi }V_{\mathrm{in}}\right)}_{11}=\sqrt{P_{\varphi }}e^{i\gamma_{\varphi }},$$

where $P_{\varphi }={\left|{\left(V_{\mathrm{out}}U_{\varphi }V_{\mathrm{in}}\right)}_{11}\right|}^{2}$ is the probability that a single photon injected in the first port is detected at the first output port of the overall network, and $\gamma_{\varphi }=\arg\left({\left(V_{\mathrm{out}}U_{\varphi }V_{\mathrm{in}}\right)}_{11}\right)$ is the phase acquired by the probe during the interferometric evolution. The Gaussian nature of the probe and of the homodyne measurements yield a Gaussian probability density function

(5) $$p_{\varphi }\left(x\right)=\frac{1}{\sqrt{2\pi \sigma_{\varphi }^{2}}}e^{-\frac{x^{2}}{2\sigma_{\varphi }^{2}}}$$

which governs the outcomes of the homodyne detection [18,19,20,21]. The univariate Gaussian probability density function in Equation (5) is centred at zero due to the absence of a displacement in the probe, while its variance is given by (see Appendix A)

(6) $$\sigma_{\varphi }^{2}=\frac{1-P_{\varphi }}{2}+\frac{P_{\varphi }}{2}\left[\cosh\left(2r\right)+\cos\left(2\gamma_{\varphi }-2\theta \right)\sinh\left(2r\right)\right],$$

where θ is the phase of the local oscillator.

Once the probability density function $p_{\varphi }\left(x\right)$ is known, it is possible to evaluate the Fisher information [44,45]

(7) $$F\left(\varphi \right)=\int_{R}dx\;p_{\varphi }\left(x\right)\frac{d}{d\varphi }\ln p_{\varphi }\left(x\right)$$

of the estimation scheme, which in turn fixes the ultimate precision $\delta \varphi_{\min}$ achievable in the estimation of φ through ν iteration of the measurement, given by the Cramer-Rao bound [44,45]

(8) $$\delta \varphi_{\min}=\frac{1}{\sqrt{\nu F(\varphi )}}.$$

For a Gaussian distribution centred on zero, the Fisher information reads (see Appendix B)

(9) $$F\left(\varphi \right)=\frac{1}{2}{\left(\frac{\partial_{\varphi }\sigma_{\varphi }^{2}}{\sigma_{\varphi }^{2}}\right)}^{2},$$

where $\partial_{\varphi }:=d/d\varphi$. We notice from Equation (6) that all the information on the parameter φ is encoded in the variance $\sigma_{\varphi }^{2}$ of the measured quadrature ${\hat{x}}_{\theta }$ through the two quantities $P_{\varphi }$ and $\gamma_{\varphi }$. Thus, we can split $\partial_{\varphi }\sigma_{\varphi }^{2}$ into two contributions, one containing the derivative of $P_{\varphi }$, the other the derivative of $\gamma_{\varphi }$, namely

(10) $$\partial_{\varphi }\sigma_{\varphi }^{2}=\left(\partial_{\varphi }P_{\varphi }\right)\partial_{P}\sigma_{\varphi }^{2}+\left(\partial_{\varphi }\gamma_{\varphi }\right)\partial_{\gamma }\sigma_{\varphi }^{2},$$

where $\partial_{P}$ and $\partial_{\gamma }$ are derivatives with respect to $P_{\varphi }$ and $\gamma_{\varphi }$, respectively, so that

(11) $$\begin{array}{cc} \partial_{P}\sigma_{\varphi }^{2} & =\frac{1}{2}\left(-1+\cosh\left(2r\right)+\cos\left(2\gamma_{\varphi }-2\theta \right)\sinh\left(2r\right)\right)\\ \partial_{\gamma }\sigma_{\varphi }^{2} & =-P_{\varphi }\sin\left(2\gamma_{\varphi }-2\theta \right)\sinh\left(2r\right). \end{array}$$

### 2.2. Heisenberg Scaling

Generally, without imposing any condition on the setup, this model does not achieve the Heisenberg scaling in the precision for the estimation of φ. In fact, we can explicitly rewrite the variance $\sigma_{\varphi }^{2}$ in Equation (6) in terms of the average number of photons $N=\sinh^{2}r$ in the probe

(12) $$\begin{array}{cc} \sigma_{\varphi }^{2} & =\frac{1-P_{\varphi }}{2}+\frac{P_{\varphi }}{2}\left[1+2N+2\cos\left(2\gamma_{\varphi }-2\theta \right)\sqrt{N(N+1)}\right]\\ \; & =NP_{\varphi }\left(1+\cos\left(2\gamma_{\varphi }-2\theta \right)\right)+O\left(1\right), \end{array}$$

where O(1) is a term of order equal to or smaller than 1, negligible in the asymptotic regime of *N* large (We will say that given two functions f(N) and g(N), f(N)=O(g(N)) when $\lim_{N\to \infty }\left|f(N)/g(N)\right|<+\infty$). We can also rewrite the derivatives $\partial_{P}\sigma_{\varphi }^{2}$ and $\partial_{\gamma }\sigma_{\varphi }^{2}$ in terms of *N*

(13) $$\begin{array}{cc} \partial_{P}\sigma_{\varphi }^{2} & =(N+\cos\left(2\gamma_{\varphi }-2\theta \right)\sqrt{N(N+1)})\\ \; & =N\left(1+\cos\left(2\gamma_{\varphi }-2\theta \right)\right)+O\left(1\right)\\ \partial_{\gamma }\sigma_{\varphi }^{2} & =-2P_{\varphi }\sin\left(2\gamma_{\varphi }-2\theta \right)\sqrt{N(N+1)}\\ \; & =-2NP_{\varphi }\sin\left(2\gamma_{\varphi }-2\theta \right)+O\left(1\right). \end{array}$$

Plugging the asymptotics shown in Equations (12) and (13) into the expression of the Fisher information in Equation (9), we notice that the numerator of F(φ) can be of the order $N^{2}$ at most. Since the denominator is in general of the order $N^{2}$ as well, it yields an overall general scaling of the Fisher information of O(1)—i.e., even lower than the SQL.

In order for this setup to reach the Heisenberg scaling, we thus need to impose some constraints which prevent the denominator of the Fisher information, i.e., the variance $\sigma_{\varphi }^{2}$, to grow with *N*. We show in Appendix C that the asymptotic conditions (Given a function f(N) and a finite sum p(N) of powers of *N*, we will say that f(N)∼p(N) when they show the same asymptotic behaviour. In formulas, f(N)∼p(N) when $f\left(N\right)=p\left(N\right)+O\left(N^{s-\varepsilon }\right)$, ∀ε>0, with *s* exponent of the smallest power of *N* appearing in the sum p(N))

(14) $$\begin{array}{cc} \gamma_{\varphi }-\theta & ∼\pm \frac{\pi }{2}+\frac{k}{N}\;k\neq 0 \end{array}$$

(15) $$\begin{array}{cc} P_{\varphi } & ∼1-\frac{\ell }{N}\;0\leq \ell <N \end{array}$$

need to be satisfied for large *N*, with 0≤ℓ<N and k≠0 arbitrary constant independent of *N*. We will discuss more in detail the physical meaning of these conditions in Section 2.3, and we will see that Equation (14) is a minimum-resolution requirement on the tuning of the local oscillator, while Equation (15) is the condition on the refocusing of the probe. Intuitively, Equations (14) and (15) assure that $\sigma_{\varphi }^{2}$ at the denominator of F(φ) does not grow as fast as $\partial_{\varphi }\sigma_{\varphi }^{2}$ at the numerator: instead, we can see in Appendix C that, when these conditions hold, the variance $\sigma_{\varphi }^{2}$ becomes of the order 1/N, while its derivative $\partial_{\varphi }\sigma_{\varphi }^{2}$ remains constant for large *N*. In particular, we show in Appendix C that the Fisher information in Equation (9) asymptotically reads

(16) $$F\left(\varphi \right)∼8\varrho \left(k,\ell \right){\left(\partial_{\varphi }\gamma_{\varphi }\right)}^{2}N^{2},$$

proving the achievement of the Heisenberg scaling, with

(17) $$\varrho \left(k,\ell \right)={\left(\frac{8k}{1+16k^{2}+4\ell }\right)}^{2}$$

positive factor reaching its maximum value for k=±1/4 and ℓ=0, namely ϱ(1/4,0)=1.

Compared with the conditions found in the literature for single-parameter Gaussian estimation schemes based on squeezed-vacuum probes which, adjusted to the notation employed so far, can be translated into $\gamma_{\varphi }-\theta =\tan^{-1}e^{2r}∼\pi /2+{\left(4N\right)}^{-1}$ and $P_{\varphi }=1$ [26,27], we see that Equations (14) and (15) achieve two important further results

- It is possible to loosen the optimal conditions found in literature, which still allow us to reach the Heisenberg scaling, at the price of a multiplying factor 0<ϱ(k,ℓ)≤1 which does not depend on *N* and hence does not ruin the scaling of the precision;
- These conditions are explicitly expressed in terms of the average number *N* of photons in the probe and, therefore, in terms of the precision we want to achieve. In Section 2.3, we will discuss how this allows us to assess the precision needed to engineer suitable auxiliary stages $V_{\mathrm{in}}$ and $V_{\mathrm{out}}$ to reach the Heisenberg scaling, showing that it is possible to avoid an iterative adaptation of the optical network.

Lastly, we recall that it is always possible to asymptotically saturate the Cramér-Rao bound in Equation (8) in the limit ν→+∞ of samples with a large number ν of observations. In particular, the maximum-likelihood estimator ${\tilde{\varphi }}_{\mathrm{MLE}}$ is an asymptotically efficient and Gaussian estimator which can be obtained through the maximisation of the Likelihood function

(18) $$L\left(\varphi ;x\right)=\prod_{i=1}^{\nu }p_{\varphi }\left(x_{\nu }\right)=\frac{1}{{\left(2\pi \sigma_{\varphi }^{2}\right)}^{\nu /2}}\exp\left(-\frac{{\left|x\right|}^{2}}{2\sigma_{\varphi }^{2}}\right),$$

associated with the set ${\left\{x_{i}\right\}}_{i=1,\ldots ,\nu }$ of the ν measurement outcomes of the quadrature field ${\hat{x}}_{\theta }$ [44,45]. In Appendix D we see that the non-trivial solution which maximises the Likelihood function L(φ;x) in Equation (18) is simply given by the estimator ${\tilde{\varphi }}_{\mathrm{MLE}}$ satisfying

(19) $$\sigma {\left({\tilde{\varphi }}_{\mathrm{MLE}}\right)}^{2}:=\sigma_{{\tilde{\varphi }}_{\mathrm{MLE}}}^{2}=S{\left(x\right)}^{2},$$

where $\sigma^{2}\left(\varphi \right)$ is the variance $\sigma_{\varphi }^{2}$ in Equation (6) as a function of φ, and $S{\left(x\right)}^{2}$ is the usual sample variance

(20) $$S{\left(x\right)}^{2}=\frac{1}{\nu }\sum_{i=1}^{\nu }x_{i}^{2}.$$

Generally, Equation (19) cannot be solved analytically, so that numerical methods need to be employed to find non-trivial solutions. Nevertheless, it is possible to find some exceptions, particularly for elementary functional dependencies of $P_{\varphi }$ and $\gamma_{\varphi }$ on the unknown parameter φ. For example, in the case for which $P_{\varphi }\equiv P$ is independent of φ, and the functional dependence of $\gamma \left(\varphi \right):=\gamma_{\varphi }$ on φ of the phase acquired by the probe is invertible, the function $\sigma {\left(\varphi \right)}^{2}$ in Equation (19) can be easily inverted as well, and the maximum-likelihood estimator reads

(21) $$\begin{array}{c} {\tilde{\varphi }}_{\mathrm{MLE}}\left(x\right)=\gamma^{-1}\left(\theta +\frac{1}{2}\left(2n\pi \pm \arccos(\frac{\left(2S{\left(x\right)}^{2}-1\right)-2P_{\varphi }\sinh^{2}r}{2P_{\varphi }\sinh r\cosh r})\right)\right). \end{array}$$

We can notice how, due to the presence of the cosine in $\sigma_{\varphi }^{2}$ in Equation (6), some prior knowledge on the parameter φ is required in order to correctly choose the invertibility interval for cos(2γ(φ)−2θ)—i.e., to choose the correct value of n∈N and the sign of the arccos function in Equation (21). In the next section, we will see how a classical prior knowledge of the parameter φ is required to satisfy condition (15), achievable with a prior coarse estimation reaching an uncertainty of the order of $1/\sqrt{N}$. Such prior knowledge on the parameter, for a large enough *N*, can be employed to choose the correct invertibility interval.

### 2.3. Conditions for the Heisenberg Scaling

We can see that both conditions in Equations (14) and (15) are φ-dependent, suggesting that an adaptive procedure must take place in order to employ the estimation scheme described in Section 2.1, as it is customary for ab-initio Gaussian estimation strategies [24,27,35,36,37]. However, some considerations can be made in this regard.

Condition (14) fixes the phase of the quadrature ${\hat{x}}_{\theta }$ which needs to be measured. The quantity $\gamma_{\varphi }$ is in fact the phase acquired by the squeezed vacuum during the interferometric evolution from the first input port to the first output port, and for $\gamma_{\varphi }\equiv \varphi$ Equation (14) resembles the condition $\theta =\varphi +\tan^{-1}e^{2r}$ found in the literature for single-phase estimation [24]. On the other hand, condition (14) is a looser condition to reach the Heisenberg scaling, and it puts in relation the precision with which we are able to choose the phase θ of the local oscillator—given by the resolution of the homodyne detection apparatus—with the precision achievable in the estimation of φ. In particular, it is evident how the minimum resolution for the homodyne detector required to reach an uncertainty δφ of order 1/N must be, in turn, of order 1/N. This is in agreement with the common notion in metrology for which a sensor cannot detect changes in the quantity that is being measured which are smaller than its resolution.

**Figure 3:**
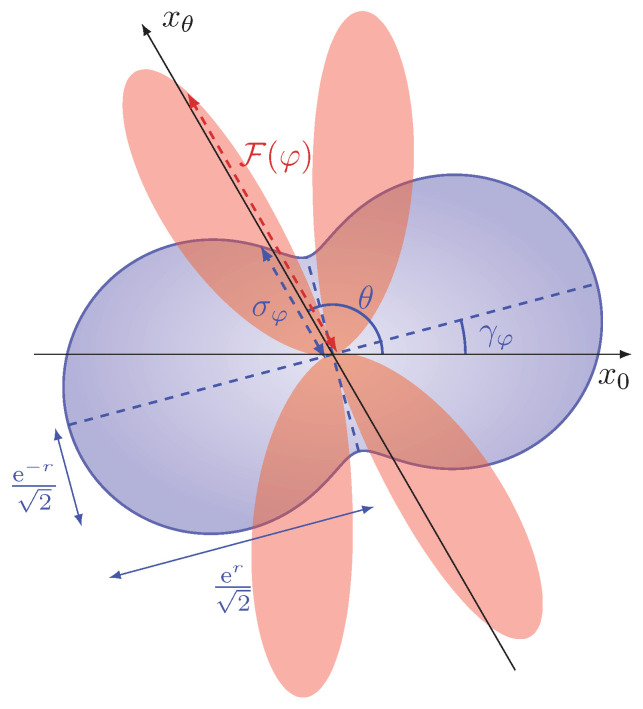
Polar plot of the standard deviation $\sigma_{\varphi }$ (see Equation (6)) in blue, and of the Fisher information F(φ) in Equation (16) in orange as functions of the phase θ of the quadrature ${\hat{x}}_{\theta }$ measured, for $P_{\varphi }=1$. The large values of F(φ) are reached for θ, satisfying condition (14). Interestingly, for $\theta =\gamma_{\varphi }\pm \pi /2$, namely when measuring the quadrature with minimum variance, $\sigma_{\varphi }$ reaches its minimum, but the Fisher information drops to zero: as a squeezing-encoding estimation scheme, this model relies on the information about φ inscribed in the variance of the quadrature measured. On the other hand, the minimum variance is a stationary point as a function of φ, and thus is locally insensitive to the variations of the parameter. Reprinted with permission from ref. [34], © 2021 The Author(s).

Interestingly, we notice from Equation (14) that the constant *k* cannot be equal to zero. Counterintuitively, the value k=0 coincides with the choice of measuring the quadrature ${\hat{x}}_{\gamma_{\varphi }+\pi /2}$, namely the minimum-variance quadrature after the squeezed vacuum undergoes a phase-shift of magnitude $\gamma_{\varphi }$, i.e., after the interferometric evolution given by ${\hat{u}}_{\varphi }$ in Equation (3). This apparent incongruity can be explained by observing the expression of $\partial_{\varphi }\sigma_{\varphi }^{2}$ in Equation (10). Differently from displacement-encoding approaches—in which the information on the parameter is obtained from the transformation of the displacement of the probe, and thus minimizing the noise of the signal is always the optimal choice—here the value of φ is encoded in the variance of the quadrature itself. For us to be able to extract information on the parameter, the variance of the signal needs to be sensitive to small variations in φ—i.e., the derivative $\partial_{\varphi }\sigma_{\varphi }^{2}$ must be non-vanishing. Of the two contributions of $\partial_{\varphi }\sigma_{\varphi }^{2}$ in Equation (10), the one originating from the variations in $P_{\varphi }$ is identically vanishing when condition (15) is satisfied, since $\partial_{\varphi }P_{\varphi }≃0$ for $P_{\varphi }$ close to its maximum. The remaining contribution derives from the variations in the overall phase $\gamma_{\varphi }$, and the variance of the maximally squeezed quadrature ${\hat{x}}_{\gamma_{\varphi }+\pi /2}$ is a stationary point with respect to variations in the phase $\gamma_{\varphi }$ and is thus insensitive to φ, namely $\partial_{\gamma }\sigma_{\varphi }^{2}=0$ for $\gamma_{\varphi }-\theta =\pi /2$ in Equation (11) (See Figure 3).

Condition (15) is the requirement that most of the photons injected into the network end up in the observed output channel. In fact, this condition can be rewritten in terms of the average number of photons that are not correctly refocused $N(1-P_{\varphi })∼\ell$. Thus, condition (15) tells us that the number of photons which are not observed must be a constant *ℓ*, not growing with *N*. In other words, this condition assures that most of the information on φ encoded in the probe is not lost in channels that are not observed. As a matter of fact, we can see from Equation (6) that the variance of the observed quadrature ${\hat{x}}_{\theta }$ after the interferometric evolution is the convex combination of the variances of a squeezed vacuum and the pure vacuum, with coefficients $P_{\varphi }$ and $1-P_{\varphi }$, respectively. In order for this variance to be ‘squeezed’, in the sense that it is of order 1/N, the contribution from the pure vacuum must be of order 1/N, namely $1-P_{\varphi }=O\left(1/N\right)$.

This condition can also be seen as a requirement of the performance of the refocusing stage ${\hat{V}}_{\mathrm{out}}$. In fact, in order to satisfy condition (15) for a given choice of ${\hat{V}}_{\mathrm{in}}$, the auxiliary stage ${\hat{V}}_{\mathrm{out}}$ must be chosen so that $|V_{\mathrm{out}}U_{\varphi }V_{\mathrm{in}}{|}^{2}∼1-\ell /N$. As discussed earlier, this implies that, in general, the auxiliary stage ${\hat{V}}_{\mathrm{out}}$ which satisfies this condition depends on the value of the parameter itself, requiring an adaptive approach to find an optimal refocusing stage to reach the Heisenberg scaling. We show now that the information on φ required to engineer an adequate refocusing stage to reach the Heisenberg scaling can be obtained through a classical estimation strategy, namely that which achieves the scaling $1/\sqrt{N}$ typical of the shot-noise limit. This result is due to the structure of $P_{\varphi }={\left|{\left(V_{\mathrm{out}}U_{\varphi }V_{\mathrm{in}}\right)}_{11}\right|}^{2}$, which is essentially a transition probability $P=|v_{\mathrm{out}}\cdot v_{\mathrm{in}}{|}^{2}$ between the unitary vectors $v_{\mathrm{in}}=U_{\varphi }V_{\mathrm{in}}e_{1}$ and $v_{\mathrm{out}}=V_{\mathrm{out}}^{†}e_{1}$, with $e_{1}={\left(1,0,\ldots ,0\right)}^{T}$. Hence, a small tilt of order $O(1/\sqrt{N})$ between the unit vectors $v_{\mathrm{in}}$ and $v_{\mathrm{out}}$ yields a quadratic reduction in their transition probability. To prove this, we will call $\varphi_{\mathrm{cl}}$ the rough guess of the value of φ that is sufficiently precise to engineer a refocusing stage ${\hat{V}}_{\mathrm{out}}$ which satisfy condition (15), and we will show that the estimation strategy to obtain this rough estimate of φ is classical, namely that the error $\delta \varphi =\varphi -\varphi_{\mathrm{cl}}$ associated with the prior rough estimation is allowed to be of order $1/\sqrt{N}$. For a given choice of ${\hat{V}}_{\mathrm{in}}$ and *ℓ*, we will call ${\hat{V}}_{\mathrm{out}}\left(\varphi_{\mathrm{cl}}\right)$ a solution of Equation (15). The single-photon transition probability $P_{\varphi }$ appearing in this condition can be written as the squared complex modulus of the scalar product of two *M*-dimensional complex vectors $U_{\varphi }V_{\mathrm{in}}e_{1}$ and $V_{\mathrm{out}}{\left(\varphi_{\mathrm{cl}}\right)}^{†}e_{1}$, with $e_{1}={\left(1,0,\ldots ,0\right)}^{T}$. We can then write

(22) $$P_{\varphi }={\left|e_{1}^{T}V_{\mathrm{out}}\left(\varphi_{\mathrm{cl}}\right)U_{\varphi }V_{\mathrm{in}}e_{1}\right|}^{2}\equiv \eta \left(\varphi ,\varphi_{\mathrm{cl}}\right),$$

where the transition probability $\eta (\varphi ,\varphi_{\mathrm{cl}})$ is a smooth function of φ and $\varphi_{\mathrm{cl}}$, with a locus of points of maxima along the condition $\varphi_{\mathrm{cl}}=\varphi$, since, for a perfect knowledge of the parameter the auxiliary stage, ${\hat{V}}_{\mathrm{out}}\left(\varphi_{\mathrm{cl}}=\varphi \right)$ would satisfy η(φ,φ)=1. If the prior estimation $\varphi_{\mathrm{cl}}$ slightly deviates from the real value of the parameter $\varphi =\varphi_{\mathrm{cl}}+\delta \varphi$, we can write the expansion

(23) $$\begin{array}{cc} P_{\varphi } & =\eta (\varphi ,\varphi -\delta \varphi )\\ \; & =1-\underset{0}{\underset{︸}{\frac{\partial \eta (\varphi ,x)}{\partial x}{|}_{x=\varphi }}}\delta \varphi +\frac{1}{2}\frac{\partial^{2}\eta \left(\varphi ,x\right)}{\partial x^{2}}{|}_{x=\varphi }\delta \varphi^{2}+O\left(\delta \varphi^{3}\right), \end{array}$$

where the derivative of $\eta (\varphi ,\varphi_{\mathrm{cl}})$ is zero along the condition $\varphi =\varphi_{\mathrm{cl}}$. We can see, comparing Equations (23) and (15), that an error δφ of order $1/\sqrt{N}$ suffices to correctly engineer a refocusing stage ${\hat{V}}_{\mathrm{out}}$ that allows for the Heisenberg scaling. It is then possible to conceive two-step ab initio protocols exploiting the model presented in this section: a first, coarse, classical estimation of the parameter φ is performed and the rough estimate $\varphi_{\mathrm{cl}}$ is obtained, with an error $\delta \varphi =\varphi -\varphi_{\mathrm{cl}}=O\left(1/\sqrt{N}\right)$ of the same order of the shot-noise limit. Then, the classical information obtained on φ can be employed to engineer the refocusing stage ${\hat{V}}_{\mathrm{out}}$, once ${\hat{V}}_{\mathrm{in}}$ is fixed, so that the overall network satisfies condition (15), allowing us to reach the Heisenberg scaling through the quantum strategy described in Section 2.1.

Lastly, we notice that, in order to satisfy condition (15), it is also possible to optimize the input auxiliary stage ${\hat{V}}_{\mathrm{in}}$ while arbitrarily fixing the refocusing stage ${\hat{V}}_{\mathrm{out}}$. In such a case, identical considerations can be made regarding the possibility of a two-step protocol, since the optimization ${\hat{V}}_{\mathrm{in}}$ still requires only a classical coarse estimation $\varphi_{\mathrm{cl}}$ of the parameter. Interestingly, only one of the auxiliary stages needs to be optimized, and thus depends on $\varphi_{\mathrm{cl}}$, whether it is ${\hat{V}}_{\mathrm{in}}$ or ${\hat{V}}_{\mathrm{out}}$. This leaves the choice of the second auxiliary stage completely arbitrary, notwithstanding that the pre-factor ${\left(\partial_{\varphi }\gamma_{\varphi }\right)}^{2}$ appearing in the Fisher information in Equation (16) is not vanishing. Indeed, the condition ${\left(\partial_{\varphi }\gamma_{\varphi }\right)}^{2}=0$ corresponds to the situation in which the optimized network ${\hat{u}}_{\varphi }={\hat{V}}_{\mathrm{out}}\left(\varphi_{\mathrm{cl}}\right){\hat{U}}_{\varphi }{\hat{V}}_{\mathrm{in}}$ acts trivially, namely without imprinting any information about φ, on the probe. Remarkably, it has been shown that, for a random choice of the non-optimized auxiliary network, sampled uniformly within the set of all the possible linear networks, the pre-factor ${\left(\partial_{\varphi }\gamma_{\varphi }\right)}^{2}$ multiplying the scaling $N^{2}$ in the Fisher information is typically different from zero [34]. In other words, within certain non-restrictive regularity conditions and for linear networks with a large enough number of channels, the value of the pre-factor becomes essentially unaffected by the choice of the non-optimised auxiliary network. This important feature can be exploited for experimental applications, for example, employing the arbitrary non-optimised stage to manipulate the information encoded into the probe regarding the structure of a linear network with multiple unknown parameters, ultimately allowing the choice of functions of such parameters to be estimated at the Heisenberg scaling sensitivity [46].

### 2.4. A Two-Channel Network

**Figure 4:**
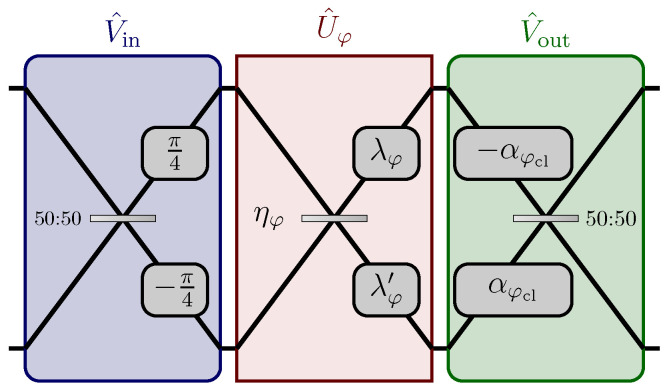
Schematic diagram of the two-channel network described in Section 2.4. The linear network ${\hat{U}}_{\varphi }$ is composed of a beam splitter with coefficient $\eta_{\varphi }$ and two phase-shifts of magnitudes $\lambda_{\varphi }$ and $\lambda_{\varphi }^{\prime }$. The auxiliary stage ${\hat{V}}_{\mathrm{in}}$ at the input is φ-independent, while the output stage ${\hat{V}}_{\mathrm{out}}$ is optimized after a classical prior estimation $\varphi_{\mathrm{cl}}$ of the parameter. In particular, the quantity $\alpha_{\varphi_{\mathrm{cl}}}=\left(\lambda_{\varphi_{\mathrm{cl}}}-\lambda_{\varphi_{\mathrm{cl}}}^{\prime }\right)/2-\pi /4$ depends on $\varphi_{\mathrm{cl}}$ only through the phase-shifts $\lambda_{\varphi_{\mathrm{cl}}}$ and $\lambda_{\varphi_{\mathrm{cl}}}^{\prime }$. Reprinted with permission from ref. [42], © 2021 The Author(s).

In this section, we will apply our model for the estimation of distributed parameters to a particular example of a 2-channel network, in which the unknown parameter φ influences the reflectivity $\eta_{\varphi }$ of a beam-splitter and the magnitudes $\lambda_{\varphi }$ and $\lambda_{\varphi }^{\prime }$ of two phase-shifts (see Figure 4). We can think of the global parameter φ as an external physical property, such as the temperature or the magnitude of the electromagnetic field, affecting the components of the network ${\hat{U}}_{\varphi }$. We will suppose that the functional dependence of the phase-shifts $\lambda_{\varphi }$, $\lambda_{\varphi }^{\prime }$ and of the reflectivity $\eta_{\varphi }$ on the true value of the parameter φ are known and smooth, whether given by some law of nature or opportunely engineered. With reference to Figure 4, we can write the matrices representing the action of the beam-splitter and the phase-shifts as

(24) $$\begin{array}{cc} U_{\mathrm{BS}}\left(\eta_{\varphi }\right) & =\exp\left(i\eta_{\varphi }\sigma_{2}\right)=\left(\begin{array}{cc} \cos\eta_{\varphi } & \sin\eta_{\varphi }\\ -\sin\eta_{\varphi } & \cos\eta_{\varphi } \end{array}\right)\\ U_{\mathrm{PS}}\left(\lambda_{\varphi },\lambda_{\varphi }^{\prime }\right) & =\exp\left(i\frac{\lambda_{\varphi }+\lambda_{\varphi }^{\prime }}{2}I_{2}+i\frac{\lambda_{\varphi }-\lambda_{\varphi }^{\prime }}{2}\sigma_{3}\right)=\left(\begin{array}{cc} \exp(i\lambda_{\varphi }) & 0\\ 0 & \exp(i\lambda_{\varphi }^{\prime }) \end{array}\right) \end{array}$$

respectively, where $\sigma_{i}$, i=1,2,3, is the *i*-th Pauli matrix and $I_{2}$ is the 2×2 identity matrix, so that the network ${\hat{U}}_{\varphi }$ is represented by the matrix

(25) $$U_{\varphi }=U_{\mathrm{PS}}\left(\lambda_{\varphi },\lambda_{\varphi }^{\prime }\right)U_{\mathrm{BS}}\left(\eta_{\varphi }\right)=\left(\begin{array}{cc} \cos\eta_{\varphi }\exp\left(i\lambda_{\varphi }\right) & \sin\eta_{\varphi }\exp\left(i\lambda_{\varphi }\right)\\ -\sin\eta_{\varphi }\exp\left(i\lambda_{\varphi }^{\prime }\right) & \cos\eta_{\varphi }\exp\left(i\lambda_{\varphi }^{\prime }\right) \end{array}\right).$$

We easily notice that $|{\left(U_{\varphi }\right)}_{11}{|}^{2}=\cos^{2}\eta_{\varphi }$, which, in general, is different from one and thus does not satisfy the condition (15), with the exception of the two values $\eta_{\varphi }=0,\pi$ which correspond to the absence of the mixing between the two modes. As described in the model earlier, we then add two auxiliary stages $V_{\mathrm{in}}$ and $V_{\mathrm{out}}\equiv V_{\mathrm{out}}\left(\varphi_{\mathrm{cl}}\right)$, of which only one depends on a prior coarse estimation $\varphi_{\mathrm{cl}}$ of φ realised with a classical strategy, so that $\varphi -\varphi_{\mathrm{cl}}=\delta \varphi =O\left(1/\sqrt{N}\right)$. In particular we choose as input stage

(26) $$V_{\mathrm{in}}=U_{\mathrm{PS}}\left(\frac{\pi }{4},-\frac{\pi }{4}\right)U_{\mathrm{BS}}\left(\frac{\pi }{4}\right)=\frac{1}{\sqrt{2}}\left(\begin{array}{cc} \exp(i\frac{\pi }{4}) & \exp(i\frac{\pi }{4})\\ -\exp(-i\frac{\pi }{4}) & \exp(-i\frac{\pi }{4}) \end{array}\right),$$

and as output stage

(27) $$V_{\mathrm{out}}=U_{\mathrm{BS}}\left(\frac{\pi }{4}\right)U_{\mathrm{PS}}\left(-\alpha_{\varphi_{\mathrm{cl}}},\alpha_{\varphi_{\mathrm{cl}}}\right)=\frac{1}{\sqrt{2}}\left(\begin{array}{cc} \exp(-i\alpha_{\varphi_{\mathrm{cl}}}) & \exp(i\alpha_{\varphi_{\mathrm{cl}}})\\ -\exp(-i\alpha_{\varphi_{\mathrm{cl}}}) & \exp(i\alpha_{\varphi_{\mathrm{cl}}}) \end{array}\right),$$

where $\alpha_{\varphi_{\mathrm{cl}}}=\left(\lambda_{\varphi_{\mathrm{cl}}}-\lambda_{\varphi_{\mathrm{cl}}}^{\prime }\right)/2-\pi /4$ is a quantity which can be obtained through a classical estimation $\varphi_{\mathrm{cl}}$ of φ. A straightforward calculation of $P_{\varphi }={\left|{\left(V_{\mathrm{out}}\left(\varphi_{\mathrm{cl}}\right)U_{\varphi }V_{\mathrm{in}}\right)}_{11}\right|}^{2}$ yields

(28) $$P_{\varphi }=\frac{1}{2}\left(1+\sin(\lambda_{\varphi }-\lambda_{\varphi }^{\prime }-2\alpha_{\varphi_{\mathrm{cl}}})\right)=\frac{1}{2}\left(1+\cos(\delta \lambda -\delta \lambda^{\prime })\right),$$

where $\delta \lambda =\lambda_{\varphi }-\lambda_{\varphi_{\mathrm{cl}}}$ and $\delta \lambda^{\prime }=\lambda_{\varphi }^{\prime }-\lambda_{\varphi_{\mathrm{cl}}}^{\prime }$ are the error in the estimates of $\lambda_{\varphi }$ and $\lambda_{\varphi }^{\prime }$ due to the imprecision of the classical estimation $\varphi_{\mathrm{cl}}$. We can then easily see that $P_{\varphi }$ in Equation (28) satisfies condition (15), since both the errors δλ and $\delta \lambda^{\prime }$ are of order $1/\sqrt{N}$,—i.e., $\delta \lambda =\left(\partial_{\varphi }\lambda_{\varphi }\right)\;\delta \varphi =O\left(1/\sqrt{N}\right)$, and similarly for $\delta \lambda^{\prime }$—and thus we obtain from Equation (28)

(29) $$P_{\varphi }∼1-\frac{1}{4}{\left(\partial_{\varphi }\lambda_{\varphi }-\partial_{\varphi }\lambda_{\varphi }^{\prime }\right)}^{2}\delta \varphi^{2}.$$

In order to evaluate the Fisher information in Equation (16), we need to calculate both the phase acquired by the probe throughout the whole interferometric evolution $\gamma_{\varphi }$, and the coefficient *ℓ*. The phase $\gamma_{\varphi }$ is easily obtained as the complex phase of ${\left(u_{\varphi }\right)}_{11}\equiv {\left(V_{\mathrm{out}}\left(\varphi_{\mathrm{cl}}\right)U_{\varphi }V_{\mathrm{in}}\right)}_{11}$

(30) $$\gamma_{\varphi }=\frac{\lambda_{\varphi }+\lambda_{\varphi }^{\prime }}{2}+\eta_{\varphi }+\frac{\pi }{2}.$$

Since $\delta \varphi =O(1/\sqrt{N})$, we call *h* the finite *N*-independent constant such that $\delta \varphi ∼h/\sqrt{N}$. The transition probability $P_{\varphi }$ can then be written as

(31) $$P_{\varphi }∼1-\frac{h^{2}}{4}{\left(\partial_{\varphi }\lambda_{\varphi }-\partial_{\varphi }\lambda_{\varphi }^{\prime }\right)}^{2}\frac{1}{N},$$

so that the factor $\ell =h^{2}{\left(\partial_{\varphi }\lambda_{\varphi }-\partial_{\varphi }\lambda_{\varphi }^{\prime }\right)}^{2}/4$ appearing in the Fisher information in Equation (16) is easily evaluated comparing Equations (15) and (31). The Fisher information can be obtained from Equation (16), with $\partial_{\varphi }\gamma_{\varphi }$ given by Equation (30), and ϱ(k,ℓ) given by Equation (17), with *k* given by the condition on the local oscillator phase and $\ell =h^{2}{\left(\partial_{\varphi }\lambda_{\varphi }-\partial_{\varphi }\lambda_{\varphi }^{\prime }\right)}^{2}/4$.

We notice from the expression of $\alpha_{\varphi_{\mathrm{cl}}}=\left(\lambda_{\varphi_{\mathrm{cl}}}-\lambda_{\varphi_{\mathrm{cl}}}^{\prime }\right)/2-\pi /4$ that the unknown reflectivity of the beam splitter $\eta_{\varphi }$ does not influence the refocusing stage ${\hat{V}}_{\mathrm{out}}\left(\varphi_{\mathrm{cl}}\right)$, but it appears in the phase $\gamma_{\varphi }$ acquired by the probe in Equation (30). In particular, if the two phases $\lambda_{\varphi }$ and $\lambda_{\varphi }^{\prime }$ are vanishing, the dependence of $\alpha_{\varphi_{\mathrm{cl}}}$, and thus of the refocusing stage ${\hat{V}}_{\mathrm{out}}\left(\varphi_{\mathrm{cl}}\right)$, on the classical estimation $\varphi_{\mathrm{cl}}$ of the parameter disappears completely. In other words, this network for $\lambda_{\varphi }=\lambda_{\varphi }^{\prime }=0$ transforms the reflectivity $\eta_{\varphi }$ of a beam splitter into the magnitude of a phase shift, independently from $\eta_{\varphi }$.

## 3. Quantum Estimation Based on Multi-Homodyne Measurements

**Figure 5:**
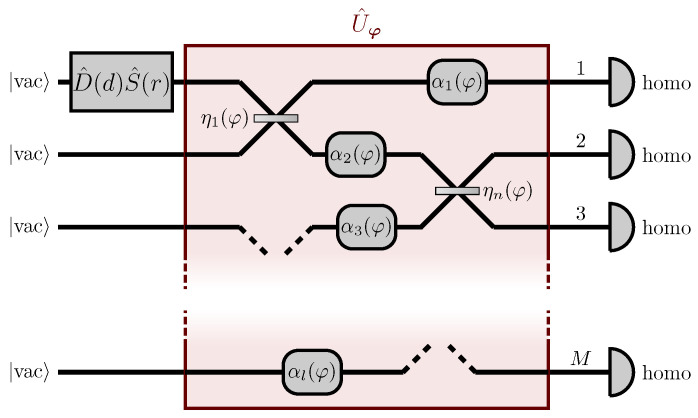
Scheme of the setup described in Section 3. A squeezed coherent state is injected in the first input port of a network ${\hat{U}}_{\varphi }$ which depends on a parameter φ that is generally distributed among multiple components of the network. Homodyne detection is performed at each of the output ports. Differently from the setup in Figure 2, no auxiliary stage is required to reach the Heisenberg scaling. Reprinted with permission from ref. [43], © 2022 The Author(s).

In Section 2 we have presented a scheme for the estimation of a distributed parameter encoded in a multi-channel network, reaching the Heisenberg scaling employing a squeezed vacuum state and homodyne detection performed at a single output channel. In particular, in Section 2.2, we have discussed in depth about the conditions in Equations (14) and (15) which need to be satisfied to reach the Heisenberg scaling: Equation (14) imposes a minimum resolution in tuning the local oscillator phase, in order to infer the value of the parameter from the noise of a sufficiently squeezed quadrature. Equation (15) is a requirement on the refocusing of the probe into the only observed channel and, in order to be satisfied, a classical knowledge of the parameter is generally required to engineer the optimal refocusing network. A natural question that arises is whether it is possible to ease these conditions by carrying out some changes on our scheme. In particular, what would happen if homodyne measurements were performed, not only at a single channel, but at all the output ports of the network instead? Would condition (15) become looser, allowing us to engineer the optimal auxiliary stages with even less information on the unknown parameter? Moreover, as we have already discussed in Section 1, a non-vanishing displacement in the probe would make it possible to infer the value of the parameter directly from the average of the quadrature with minimum variance, and not from its noise, which may be a more feasible approach in particular experimental scenarios. In this section, we will investigate a model that implements these changes (see Figure 5). We will see that, with these assumptions, not only is there no need to perform a prior estimation of the parameter to optimize the network, but the Heisenberg scaling can be achieved without employing an auxiliary stage in the first place. Moreover, the presence of displacement in the probe will allow us to perform the estimation directly measuring the minimum-variance quadrature, relying on the information about the parameter encoded in the average signal of the homodyne, and not in its noise.

### 3.1. Setup

We will consider a linear and passive network ${\hat{U}}_{\varphi }$, which depends on a single and generally distributed parameter, for example affecting several components of the network, as shown in Figure 1. Once again, ${\hat{U}}_{\varphi }$ admits a unitary matrix representation $U_{\varphi }$ obtained through Equation (1). Differently from Section 2 though, we consider as a probe the single-mode squeezed state

(32) $$|\psi_{0}\rangle =|\alpha ,r\rangle \equiv \hat{D}\left(\alpha \right)\hat{S}\left(r\right)|\mathrm{vac}\rangle ,\;\alpha =\left(\alpha ,0,\ldots ,0\right),\;r=\left(r,0,\ldots ,0\right),$$

with $\hat{D}\left(\alpha \right)=e^{\alpha ({\hat{a}}_{1}^{†}-{\hat{a}}_{1})}$, i.e., a squeezed coherent state with a mean number of photons $N=N_{D}+N_{S}=\alpha^{2}+\sinh^{2}r$, α,r>0, where we can introduce the displacement d, given by $\alpha_{i}=\left(d_{i}+id_{i+M}\right)/\sqrt{2}$, i=1,…,M. We remark that the choice α,r>0 is a specific (φ-independent) condition which is required in displacement-encoding approaches: squeezing and displacement can, in general, have different complex phases, and the condition α,r>0 assures that the squeezed quadrature (${\hat{x}}_{\pi /2}$ for r>0) is orthogonal—i.e., conjugated—to the displaced quadrature (${\hat{x}}_{0}$ for α>0), and hence it is the most sensible to the presence of phases. Moreover, in our model, we will consider homodyne detection in all *M* output channels of the linear network, so that *M* quadrature fields ${\hat{x}}_{i,\theta_{i}}$ are measured, where $\theta_{i}$ is the phase of the *i*-th local oscillator, i=1,…,M.

Since we are observing all the output ports of the network, but a non-vanishing number of photons is injected only in the first channel, only the first column of the unitary matrix $U_{\varphi }$ is relevant in our model, consisting of the transition amplitude of single photons from the first to every channel of the network (see Equation (1)). We can thus employ the parametrisation

(33) $${\left(U_{\varphi }\right)}_{i1}=\sqrt{P_{i}}e^{i\gamma_{i}},$$

where $P_{i}$ is the probability that a single photon is transmitted through the linear network from the first to the *i*-th channel, and $\gamma_{i}$ is the phase that it would acquire (In order to keep the notation lighter, we are dropping the subscript φ in $P_{i}$, $\gamma_{i}$, μ and Σ. Nonetheless, it rests assured that these quantities, in general, depend on the unknown parameter). Once again, due to the Gaussian nature of the model, the (joint) probability distribution associated with the outcome x of the *M* homodyne detectors is also Gaussian and reads [18,19,20,21]

(34) $$p_{\varphi }\left(x\right)=\frac{1}{\sqrt{{\left(2\pi \right)}^{M}\det\left[\Sigma \right]}}\exp\left(-\frac{1}{2}{\left(x-\mu \right)}^{T}\Sigma^{-1}\left(x-\mu \right)\right).$$

In Equation (34), both the covariance matrix Σ and the mean μ depend on the parameter φ. The elements of the covariance matrix Σ are evaluated in Appendix A, and read

(35) $${\left(\Sigma \right)}_{ij}=\frac{\delta_{ij}}{2}+\sqrt{P_{i}P_{j}}\left(\cos\left({\bar{\gamma }}_{i}-{\bar{\gamma }}_{j}\right)\sinh{\left(r\right)}^{2}+\cos\left({\bar{\gamma }}_{i}+{\bar{\gamma }}_{j}\right)\cosh\left(r\right)\sinh\left(r\right)\right),$$

where $\delta_{ij}$ is the Kronecker delta, ${\bar{\gamma }}_{i}=\gamma_{i}-\theta_{i}$ is the phase acquired at the output of the *i*-th channel relative to the correspondent local oscillator, and μ is the mean vector (see Appendix A)

(36) $$\mu_{i}=d\sqrt{P_{i}}\cos{\bar{\gamma }}_{i},\;i=1,\ldots ,M.$$

The determinant det[Σ] can also be written in compact form (see Appendix A)

(37) $$\begin{array}{cc} \det\left[\Sigma \right]=\frac{1}{2^{M}} & +\frac{\sinh(r)}{2^{M-1}}\sum_{i=1}^{M}P_{i}\left(\sinh\left(r\right)+\cos\left(2{\bar{\gamma }}_{i}\right)\cosh\left(r\right)\right)\\ \; & -\frac{\sinh^{2}\left(r\right)}{2^{M-2}}\sum_{i=1}^{M}\sum_{j=i+1}^{M}P_{i}P_{j}\sin^{2}\left({\bar{\gamma }}_{i}-{\bar{\gamma }}_{j}\right). \end{array}$$

For a multivariate Gaussian distribution of the form shown in Equation (34), the Fisher information can be easily evaluated from its definition in Equation (7) and employ the expression of the probability distribution $p_{\varphi }\left(x\right)$ in Equation (34) as (see Appendix B)

(38) $$\begin{array}{c} F\left(\varphi \right)=\underset{F_{D}\left(\varphi \right)}{\underset{︸}{\frac{1}{\det[\Sigma ]}\partial_{\varphi }\mu^{T}C\partial_{\varphi }\mu }}+\underset{F_{S}\left(\varphi \right)}{\underset{︸}{\frac{1}{2}{\left(\frac{\partial_{\varphi }\det\left[\Sigma \right]}{\det[\Sigma ]}\right)}^{2}-\frac{1}{2\det[\Sigma ]}\left[\left(\partial_{\varphi }\Sigma \right)\left(\partial_{\varphi }C\right)\right]}} \end{array}$$

where $C=\det\left[\Sigma \right]\Sigma^{-1}$ is the cofactor matrix of Σ and $\left[A\right]=\sum_{i}A_{ii}$ denotes the trace of the matrix *A*. Compared with the Fisher information shown in Equation (9) for the model described in Section 2, the Fisher information for this setup includes an additional term $F_{D}\left(\varphi \right)$ which depends on the derivative of the average μ with respect to the parameter φ. Moreover, the contribution $F_{S}\left(\varphi \right)$, representing the information on φ encoded in the covariance matrix Σ, can be split into two terms, of which the first resembles the Fisher information in Equation (9) once we perform the substitution $\sigma_{\varphi }^{2}\to \det\left[\Sigma \right]$: we will show in the following that, in the asymptotic regime of large $N_{S}$, this is the only contribution of $F_{S}\left(\varphi \right)$ which, besides $F_{D}\left(\varphi \right)$, reaches the Heisenberg scaling.

### 3.2. Heisenberg Scaling

Similarly to what happens to the setup described in Section 2, the Fisher information in Equation (38) does not generally reach the Heisenberg scaling unless certain conditions are met. To show this, it is necessary to evaluate all the contributions of F(φ) in terms of the number of photons $N_{D}$ and $N_{S}$ in the asymptotic regime. First, it is convenient to express the cofactor matrix *C* explicitly in terms of the squeezing parameter *r*. In Appendix B, we see that a closed form for *C* in such terms exists

(39a) $$\begin{array}{cc} C_{ss} & =\frac{1}{2^{M-1}}+\frac{1}{2^{M-2}}\sum_{\begin{array}{c} i=1\\ i\neq s \end{array}}^{M}\left(\Sigma_{ii}-\frac{1}{2}\right)-\frac{1}{2^{M-3}}\sum_{\begin{array}{c} i=1\\ i\neq s \end{array}}^{M}\sum_{\begin{array}{c} j=i+1\\ j\neq s \end{array}}^{M}S_{iij}, \end{array}$$

(39b) $$\begin{array}{cc} C_{st} & =-\frac{1}{2^{M-2}}\Sigma_{st}+\frac{1}{2^{M-3}}\sum_{\begin{array}{c} i=1\\ i\neq s,t \end{array}}^{M}S_{sti}\;,\;s\neq t, \end{array}$$

where

(40) $$S_{sti}=\sinh^{2}r\sqrt{P_{s}P_{t}}P_{i}\sin\left(\gamma_{s}-\gamma_{i}\right)\sin\left(\gamma_{t}-\gamma_{i}\right),$$

and $\Sigma_{ij}$ are shown in Equation (35). We then notice that, in order to analyse the generic asymptotic behaviour of the Fisher information in Equation (38), it suffices to separately examine the asymptotics of the terms μ, $\Sigma_{ij}$, $S_{sti}$, det[Σ], and of their derivatives.

From Equations (35)–(37), (39) and (40) we see that Σ, *S* and det[Σ] are all of order $N_{S}$ in general, while μ is of order $\sqrt{N_{D}}$. The same asymptotic behaviours are also kept for their respective derivatives, since both $\partial_{\varphi }{\bar{\gamma }}_{i}\equiv \partial_{\varphi }\gamma_{i}$ and $\partial_{\varphi }P_{i}$ are independent of the probe, and thus of $N_{D}$ and $N_{S}$. We can thus see from the expression of F(φ) in Equation (38) that its numerator grows at most with $N^{2}$, while the denominator—i.e., the denominator det[Σ]—in general grows with $N_{S}$. Therefore, in order for F(φ) to reach the Heisenberg scaling, namely a scaling of order $N^{2}$, some conditions must be imposed so that the denominators in $F_{D}\left(\varphi \right)$ and $F_{S}\left(\varphi \right)$ do not grow for large $N_{S}$. In Appendix C, it can be seen that, to achieve the Heisenberg scaling, the determinant det[Σ] must be of the order $N^{-1}$, similarly to what happens to $\sigma_{\varphi }^{2}$ in the single-homodyne setup in Section 2.2, and the conditions for this to occur are

(41) $${\bar{\gamma }}_{i}=\pm \frac{\pi }{2}+O\left(N_{S}^{-1}\right),\;i=1,\ldots ,M.$$

When these conditions hold, we can introduce the finite (possibly vanishing) quantities $k_{i}=\lim_{N_{S}\to \infty }N_{S}\left(\gamma_{i}\mp \pi /2\right)$, so that the determinant det[Σ] reduces to (see Appendix C)

(42) $$\det\left[\Sigma \right]∼\frac{1}{2^{M-2}N_{S}}\left({\left(\sum_{i=1}^{M}P_{i}k_{i}\right)}^{2}+\frac{1}{16}\right),$$

while $\partial_{\varphi }\det\left[\Sigma \right]$, $\partial_{\varphi }\Sigma$, $\partial_{\varphi }C$ and *C* tend to constant values, and $\partial_{\varphi }\mu$ scales as $\sqrt{N_{D}}$. We can easily see that this makes only the first two terms of F(φ) in Equation (38) dominant for large $N_{D}$ and $N_{S}$. When conditions (41) are met, we can thus neglect the last term in Equation (38), and the Fisher information

(43) $$\begin{array}{cc} F(\varphi ) & ∼\frac{1}{\det[\Sigma ]}\partial_{\varphi }\mu^{T}C\partial_{\varphi }\mu +\frac{1}{2}{\left(\frac{\partial_{\varphi }\det\left[\Sigma \right]}{\det[\Sigma ]}\right)}^{2}\\ \; & ∼8{\left(\partial \gamma \right)}_{\mathrm{avg}}^{2}\left(\zeta \left(k_{\mathrm{avg}}\right)2N_{D}N_{S}+\varrho \left(k_{\mathrm{avg}}\right)N_{S}^{2}\right), \end{array}$$

asymptotically reaches the Heisenberg scaling in $N_{D}N_{S}$ and $N_{S}^{2}$ (see Appendix C), where we introduced the quantities

(44) $$k_{\mathrm{avg}}\equiv \sum_{i=1}^{M}P_{i}k_{i},\;{\left(\partial \gamma \right)}_{\mathrm{avg}}\equiv \sum_{i=1}^{M}P_{i}\partial_{\varphi }\gamma_{i},$$

while

(45) $$\begin{array}{c} \zeta \left(x\right)={\left(16x^{2}+1\right)}^{-1},\;\varrho \left(x\right)={\left(8x\right)}^{2}/{\left(16x^{2}+1\right)}^{2} \end{array}$$

are positive, even functions which reach their maxima at x=±1/4 and x=0, respectively, namely ϱ(1/4)=1 and ζ(0)=1.

It is now possible to compare the Fisher information in Equation (43) achieved with the present scheme, with the Fisher information in Equation (16) obtained with the setup for distributed metrology employing a squeezed vacuum state and homodyne detection on a single channel. Since this setup employs a squeezed probe with a non-vanishing displacement, it lends itself to both displacement-based and squeezing-based estimation approaches. This is reflected by the presence of two separate contributions to the Fisher information in Equation (43): the first is given by the variations in the displacement μ, the second by the variations in the determinant det[Σ], with respect to changes in the value of φ. Both terms present a pre-factor ${\left(\partial \gamma \right)}_{\mathrm{avg}}^{2}$ shown in Equation (44) which resembles the pre-factor ${\left(\partial_{\varphi }\gamma_{\varphi }\right)}^{2}$ in Equation (16) for the single-homodyne counterpart: in particular, ${\left(\partial \gamma \right)}_{\mathrm{avg}}^{2}$ is a weighted average of the sensitivities of the complex phases $\gamma_{i}$ to changes in the parameter φ, each one weighted by the ‘fraction’ $P_{i}$ of the probe undergoing the phase shift $\gamma_{i}$. Indeed, we can see that ${\left(\partial \gamma \right)}_{\mathrm{avg}}^{2}$ reduces to the single-homodyne counterpart when the whole probe is refocused into a single channel—say the first—so that $P_{1}=1$ and $P_{i}=0$ for i=2,…,M, i.e., ${\left(\partial \gamma \right)}_{\mathrm{avg}}^{2}={\left(\partial_{\varphi }\gamma_{1}\right)}^{2}$.

In the term of the Fisher information associated with the squeezing-encoding in Equation (43), the factor $\varrho (k_{\mathrm{avg}})$ coincides in turn with the function ϱ(k,ℓ) in Equation (17) for ℓ=0—i.e., the ideal case of perfect refocusing of the probe, and all photons being observed—and after the substitution $k\to k_{\mathrm{avg}}$, namely the weighted average of the coefficients $k_{i}$ with the same weights $P_{i}$. In other words, this term can be thought of as a generalisation of the Fisher information for a single homodyne due to the presence of multiple observed channels. Noticeably, this term can be set as equal to zero only for $N_{S}=0$, in which case, the whole expression in Equation (43) vanishes, ruining the Heisenberg scaling, or for $k_{\mathrm{avg}}=0$. In fact, we can see from Equation (37) that the condition $k_{\mathrm{avg}}=0$—i.e., ${\bar{\gamma }}_{i}=\pm \pi /2$, namely when the quadratures with minimum variances are measured at each channel minimises det[Σ], which becomes a stationary point with respect to the variations in φ, and $\partial_{\varphi }\det\left[\Sigma \right]$ in Equation (43) vanishes. On the other hand, the first contribution to Equation (43) is instead a new term not present in the Fisher information for the single homodyne, deriving from the information on the parameter encoded in the displacement μ. This term achieves the Heisenberg scaling in $N_{D}N_{S}$, in the sense that it reaches the Heisenberg scaling in $N=N_{D}+N_{S}$ if both $N_{D}$ and $N_{S}$ grow with *N*, i.e., if $N_{D}=\beta N$ and $N_{S}=\left(1-\beta \right)N$ with 0≤β<1 independently of *N*. The function $\zeta (k_{\mathrm{avg}})$ in Equation (45) does not have roots, hence the first contribution to F vanishes only for $N_{D}=0$, i.e., for a squeezed vacuum as a probe.

Finally, we will now write the Likelihood function L(φ;x) for the setup described here, and discuss the maximum-likelihood estimator ${\tilde{\varphi }}_{\mathrm{MLE}}$ saturating the Cramér-Rao bound shown in Equation (8). After performing ν measurements of the field quadratures ${\hat{x}}_{i,\theta_{i}}$, the Likelihood of the outcomes $\left(x_{1},\ldots ,x_{\nu }\right)$ is given by

(46) $$L\left(\varphi ;x_{1},\ldots ,x_{\nu }\right)=\prod_{j=1}^{\nu }p_{\varphi }\left(x_{j}\right),$$

with $p_{\varphi }\left(x_{j}\right)$ found in Equation (34). In Appendix D, we show that the maximum-likelihood estimator, a non-trivial solution of the maximisation of the Likelihood function in Equation (46), is implicitly given by the estimator ${\tilde{\varphi }}_{\mathrm{MLE}}$ which satisfies the equation

(47) $$\begin{array}{cc} 0 & =\frac{1}{2}\mathrm{Tr}{\left[\partial_{\varphi }\Sigma^{-1}\left(\nu \Sigma -\sum_{j=1}^{\nu }\left(x_{j}-\mu \right){\left(x_{j}-\mu \right)}^{T}\right)\right]}_{\varphi ={\tilde{\varphi }}_{\mathrm{MLE}}}\\ \; & \;+{\left[{\left(\partial_{\varphi }\mu \right)}^{T}\Sigma^{-1}\left(\nu \mu -\sum_{j=1}^{\nu }x_{j}\right)\right]}_{\varphi ={\tilde{\varphi }}_{\mathrm{MLE}}}, \end{array}$$

where Σ is the covariance matrix in Equation (35), and μ the mean vector in Equation (36). This equation cannot, in general, be solved analytically, hence numerical methods typically need to be in place to find ${\tilde{\varphi }}_{\mathrm{MLE}}$. On the other hand, Equation (47) simplifies for certain particular cases. For example, when measuring all the minimum-variance quadratures so that ${\bar{\gamma }}_{i}=\pm \pi /2$ in Equation (41), and the probabilities $P_{i}$ are independent of φ, we can see from Equation (35) that $\partial_{\varphi }\Sigma =0$. In this case, the Likelihood Equation becomes

(48) $$0={\left[{\left(\partial_{\varphi }\mu \right)}^{T}\Sigma^{-1}\left(\mu -\frac{1}{\nu }\sum_{j=1}^{\nu }x_{j}\right)\right]}_{\varphi ={\tilde{\varphi }}_{\mathrm{MLE}}},$$

where the term in the right-hand side can be seen as a linear combination of the quantities $\mu -\tilde{\mu }$, where $\tilde{\mu }=\sum_{j=1}^{\nu }x_{j}/\nu$ are estimators of the mean μ. We see here how the displacement of the probe allows us to perform the estimation of φ through the sample mean μ, i.e., the average of the outcomes of the homodyne measurements. On the other hand, for a squeezed vacuum such as a probe, μ=0, so that Equation (47) becomes

(49) $$0=\frac{1}{2}\mathrm{Tr}{\left[\partial_{\varphi }\Sigma^{-1}\left(\Sigma -\frac{1}{\nu }\sum_{j=1}^{\nu }x_{j}x_{j}^{T}\right)\right]}_{\varphi ={\tilde{\varphi }}_{\mathrm{MLE}}},$$

where it is possible to recognise the sample covariance matrix $\tilde{\Sigma }=\sum_{j=1}^{\nu }x_{j}x_{j}^{T}/\nu$, estimator of the covariance matrix Σ.

### 3.3. Conditions for the Heisenberg Scaling

For the model introduced in this section, some considerations regarding the conditions in Equation (41) can also be drawn, especially in light of the feature of the single-homodyne, squeezed-vacuum scheme discussed in Section 2.3.

In fact, these conditions do nothing but fix the phases $\theta_{i}$ of the quadratures ${\hat{x}}_{i,\theta_{i}}$ that need to be measured to achieve the Heisenberg scaling. Regarding the minimum resolution of the homodyne sensors, it appears that there is no evident advantage in this setup compared to the single-homodyne scheme, since the resolution required to tune the local oscillators of each channel is still of order 1/N. On the other hand, introducing displacement in the probe allows, as previously discussed, the value of the parameter to be inferred from the information inscribed in the average of the measured quadratures μ. Therefore, it is possible with this scheme to exactly measure the minimum-variance quadratures ${\hat{x}}_{i,\gamma_{i}\pm \pi /2}$, a possibility that was prevented in the previous scheme due to the requirement k≠0 in Equation (14). Although the situation for which $k_{i}=0$ for i=1,…,M sets to zero the contribution $F_{S}\left(\varphi \right)$ in Equation (43), associated with the information encoded in the covariance matrix Σ, the first contribution $F_{D}\left(\varphi \right)$ of the Fisher information still reaches the Heisenberg scaling.

Another interesting feature of this protocol, which differentiates it from its single-homodyne counterpart, is that it does *not* require any adaptation of the network: since every output channel is observed, no condition on the transition probabilities $P_{i}$ is required. Therefore, not only there is no requirement for a φ-dependent auxiliary stage, but there is no need for an auxiliary stage to reach the Heisenberg scaling in the first place. However, the precision in the estimation is still affected by the network through the terms $k_{\mathrm{avg}}$ and ${\left(\partial \gamma \right)}_{\mathrm{avg}}$, which appear in the constant factors multiplying the scaling in the Fisher information in Equation (38). In fact, we can see from Equation (45) that these two quantities depend on the transition probabilities $P_{i}$ and on the derivatives of the complex phases $\partial_{\varphi }\gamma_{i}$. In particular, F(φ) can be vanishing for exceptionally poorly conceived networks, for which ${\left(\partial \gamma \right)}_{\mathrm{avg}}=0$: for example, a network for which $\gamma_{i}$ is independent of φ for all values of *i* such that $P_{i}\neq 0$, is associated with a vanishing factor ${\left(\partial \gamma \right)}_{\mathrm{avg}}$, as we can see from its definition in Equation (44). In this case, adding a φ-independent auxiliary network *V*, either at the input or at the output of $U_{\varphi }$, would modify the transition amplitude of the overall interferometric evolution, and ultimately yield a non-vanishing value of ${\left(\partial \gamma \right)}_{\mathrm{avg}}$.

## 4. Conclusions

The recent advances in quantum metrology made possible the realisation of protocols achieving super-sensitivity in the estimation of optical lengths, time delays and space-time distortions due to gravitational waves, refractive indices in given materials, density and thickness of biological samples up to the nanometer scale, temperatures, polarisations of light, magnitudes of fields and their gradients, and more. However, current quantum sensing technologies still present some limitations, whether caused by the requirement of adaptively optimising the estimation procedure to the unknown value of the parameter that needs to be measured, by the fragility of the metrological resources which yield super-sensitivity, or by the scarce operating range of the protocols, impracticalities arise when tackling the metrological schemes with quantum mechanics. Moreover, most standard approaches to distributed quantum metrology suffer from a lack of universal estimation schemes, namely those which can operate independently of the unknown parameter nature and value, and of the unitary evolution which encodes the value of the parameter into the probe employed for the estimation.

In this work, we have reviewed in detail two schemes which address these limitations [42,43]. By employing analyses based on the Cramér–Rao bound, i.e., the ultimate precision achievable for a given estimation scheme, and on the Fisher information, we were able to assess the super-sensitivity of various feasible metrological setups, always achievable in the regime of large statistical samples through the maximum-likelihood estimator. We showed that, without making any assumptions on the structure of the multi-channel passive and linear network encoding the unknown parameter, which can as well be distributed among multiple components of the interferometer (such as temperature affecting the network in a distributed manner) it is possible to relax the adaptivity of the network to a requirement of classical knowledge—i.e., not at the quantum limit—on the parameter. This prior knowledge serves to engineer an auxiliary stage needed to perform the super-sensitive estimation with a single squeezed-vacuum state and homodyne detection at a single output port. Moreover, the adaptivity on the network can actually be completely circumvented when all the output ports of the network are observed with homodyne detectors. We discussed how this allows us to conceive estimation schemes at the Heisenberg limit, which can be performed with a single optimization step, when a classical knowledge of the parameter is needed to engineer the auxiliary stage, or without any optimization at all, when no prior knowledge is required.

## Data Availability

Not applicable.

## Appendix A. Probability Distributions from Homodyne Measurements

In this appendix, we will obtain the probability density functions which govern the outcomes of homodyne detections performed on a single-mode squeezed state $|\alpha ,r\rangle =\hat{D}\left(\alpha \right)\hat{S}\left(r\right)|\mathrm{vac}\rangle$ injected in the first port of a *M*-channel passive and linear network $\hat{U}$, with $\alpha =\left(\alpha ,0,\ldots ,0\right)=\left(\sqrt{N_{D}},0,\ldots ,0\right)$, and r=(r,0,…,0), so that $N_{S}=\sinh^{2}r$, with α,r>0, and $N=N_{D}+N_{S}$ sum of the average number of photons in the displacement and in the squeezing of the state |α,r〉. We refer to refs. [18,19,20,21] for reviews on Gaussian states, and how to describe and manipulate them mathematically. The state |α,r〉 is a Gaussian state which is completely described, through its Wigner distribution

(A1) $$W\left(z\right)=\frac{1}{\sqrt{{\left(2\pi \right)}^{2M}\det\left[\Gamma \right]}}\exp\left(-\frac{1}{2}{\left(z-d\right)}^{T}\Gamma^{-1}\left(z-d\right)\right),$$

by its vector of displacement $d=\sqrt{2}\;\left(\alpha ,0,\ldots ,0\right)$, and its matrix of covariances $\Gamma =\mathrm{diag}(e^{2r},1\ldots ,1,e^{-2r},1\ldots ,1)/2$. Moreover, passive and linear networks preserve the Gaussian nature of |α,r〉. For this reason, we will first obtain in generality the final displacement $d_{U}$ and covariance matrix $\Gamma_{U}$ of the state $\hat{U}|\alpha ,r\rangle$. Then, we will marginalise the Wigner distribution, rotated by the symplectic and orthogonal matrix

(A2) $$R\left(\theta \right)=\left(\begin{array}{cc} \cos(\Theta ) & \sin(\Theta )\\ -\sin(\Theta ) & \cos(\Theta ) \end{array}\right),$$

accordingly to the phases $\Theta =\mathrm{diag}\left(\theta \right)=\mathrm{diag}\left(\theta_{1},\ldots ,\theta_{M}\right)$ of the local oscillators of the homodyne. In doing so, we will be able to obtain the expressions in Equations (6), and (35)–(37).

The action of a passive and linear unitary $\hat{U}$ on a state is described by a symplectic rotation of the state Wigner distribution [18,19,20,21]. The Wigner distribution $W_{U}\left(z\right)$ of the state $\hat{U}|\alpha ,r\rangle$ after the interferometric evolution $\hat{U}$ is thus obtained, rotating the initial Wigner distribution W(z) found in Equation (A1) with the orthogonal and symplectic matrix

(A3) R=Re(U)−Im(U)Im(U)Re(U),

where *U* is the unitary matrix representing the transition amplitudes which can be found through Equation (1), thus obtaining the transformation

(A4) $$W_{U}\left(z\right)=W\left(R^{T}z\right).$$

Since the probe in our model is Gaussian, the transformation of the state is completely described by the rotations $d_{U}=Rd$ of the displacement and $\Gamma_{U}=R\Gamma R^{T}$ of the covariance matrix of the initial state, with $\Gamma =\mathrm{diag}(e^{2R},e^{-2R})/2$, R=diag(r,0,…,0), and $d=\sqrt{2}\;\mathrm{diag}\left(\alpha ,0,\ldots ,0\right)$. A straightforward calculation shows that

(A5) dU=Rd=2αRe[U11]⋮Re[UM1]Im[U11]⋮Im[UM1]

and that

(A6) $$\Gamma_{U}=R\Gamma R^{T}=\left(\begin{array}{cc} \Delta X^{2} & \Delta XP\\ \Delta XP^{T} & \Delta P^{2} \end{array}\right).$$

where we have defined the M×M matrices

(A7a) ΔX2≡12Re[U]e2RRe[U†]−Im[U]e−2RIm[U†]=12Re[Ucosh(2R)U†]+Re[Usinh(2R)UT],

(A7b) ΔP2≡12−Im[U]e2RIm[U†]+Re[U]e−2RRe[U†]=12Re[Ucosh(2R)U†]−Re[Usinh(2R)UT],

(A7c) ΔXP≡12−Re[U]e2RIm[U†]−Im[U]e−2RRe[U†]=12−Im[Ucosh(2R)U†]+Im[Usinh(2R)UT].

We can see that the only elements of *U* entering in Equation (A5) are the ones in the first column $U_{i1}$, since these are all the transition amplitudes from the only populated input port (namely the first) to every output port. We can easily see that the same is true for the matrices in Equation (A7). In fact, exploiting the relations

(A8a) $$\begin{array}{c} \cosh{\left(2R\right)}_{ij}=\delta_{ij}+\delta_{i1}\delta_{j1}\left(\cosh2r-1\right) \end{array}$$

(A8b) $$\begin{array}{c} \sinh{\left(2R\right)}_{ij}=\delta_{i1}\delta_{j1}\sinh2r, \end{array}$$

where $\delta_{ij}$ is the Kronecker delta, we can evaluate

(A9a) $$\begin{array}{c} {\left(U\cosh\left(2R\right)U^{†}\right)}_{ij}=\sum_{k,l=1}^{M}U_{ik}\cosh{\left(2R\right)}_{kl}U_{lj}^{†}=\delta_{ij}+U_{i1}U_{j1}^{*}\left(\cosh2r-1\right) \end{array}$$

(A9b) $$\begin{array}{c} {\left(U\sinh\left(2R\right)U^{T}\right)}_{ij}=\sum_{k,l=1}^{M}U_{ik}\sinh{\left(2R\right)}_{kl}U_{lj}^{T}=U_{i1}U_{j1}\sinh2r. \end{array}$$

We can thus parametrise only the first column of *U*, introducing the quantities $P_{i}={\left|U_{i1}\right|}^{2}$ and $\gamma_{i}=\arg U_{i1}$ for i=1,…,M, which are respectively the transition probabilities from the first input to the *i*-th output port, and the complex phases of the relevant transition amplitudes, so that $U_{i1}=\sqrt{P_{i}}e^{i\gamma_{i}}$. We can then rewrite the displacement $d^{\prime }$ in Equation (A5) and the covariance matrix $\Gamma^{\prime }$ in Equation (A6) in terms of $P_{i}$ and $\gamma_{i}$

(A10) $$d_{U}=Rd=\sqrt{2}\alpha \left(\begin{array}{c} \sqrt{P_{1}}\cos\gamma_{1}\\ \vdots \\ \sqrt{P_{M}}\cos\gamma_{M}\\ \sqrt{P_{1}}\sin\gamma_{1}\\ \vdots \\ \sqrt{P_{M}}\sin\gamma_{M} \end{array}\right),$$

and

(A11a) $$\begin{array}{cc} \Delta X_{ij}^{2} & =\frac{1}{2}\delta_{ij}+\sqrt{P_{i}P_{j}}\left(\cos\left(\gamma_{i}-\gamma_{j}\right)\sinh^{2}r+\cos\left(\gamma_{i}+\gamma_{j}\right)\sinh r\cosh r\right) \end{array}$$

(A11b) $$\begin{array}{cc} \Delta P_{ij}^{2} & =\frac{1}{2}\delta_{ij}+\sqrt{P_{i}P_{j}}\left(\cos\left(\gamma_{i}-\gamma_{j}\right)\sinh^{2}r-\cos\left(\gamma_{i}+\gamma_{j}\right)\sinh r\cosh r\right) \end{array}$$

(A11c) $$\begin{array}{cc} \Delta XP_{ij} & =\sqrt{P_{i}P_{j}}\left[-\sin\left(\gamma_{i}-\gamma_{j}\right)\sinh^{2}r+\sin\left(\gamma_{i}+\gamma_{j}\right)\sinh r\cosh r\right]. \end{array}$$

Finally, we notice that if we are performing homodyne to measure the quadratures ${\hat{x}}_{i,\theta_{i}}$, where $\theta =(\theta_{1},\ldots ,\theta_{M})$ are the phases of the local oscillators at each output channel, this corresponds to apply a further unitary rotation $U\left(\theta \right)=\mathrm{diag}\left(e^{-i\theta_{1}},\ldots ,e^{-i\theta_{M}}\right)$ on the state U|α,r〉. This corresponds to substituting U→U(θ)U in the expression of *R* in Equation (A3), whose effect is to add a phase $e^{-i\theta_{i}}$ to every elements of the *i*-th row of *U*. Thus, if we define ${\bar{\gamma }}_{i}=\gamma_{i}-\theta_{i}$, we can write the displacement $d_{\theta }$ and the covariance matrix $\Gamma_{\theta }$ of the (accessory) state $\hat{U}\left(\theta \right)\hat{U}|\alpha ,d\rangle$

(A12) $$d_{\theta }=\sqrt{2}\alpha \left(\begin{array}{c} \sqrt{P_{1}}\cos{\bar{\gamma }}_{1}\\ \vdots \\ \sqrt{P_{M}}\cos{\bar{\gamma }}_{M}\\ \sqrt{P_{1}}\sin{\bar{\gamma }}_{1}\\ \vdots \\ \sqrt{P_{M}}\sin{\bar{\gamma }}_{M} \end{array}\right),$$

and

(A13) $$\Gamma_{\theta }=\left(\begin{array}{cc} \Delta X_{\theta }^{2} & \Delta XP_{\theta }\\ \Delta XP_{\theta }^{T} & \Delta P_{\theta }^{2} \end{array}\right),$$

with

(A14a) $$\begin{array}{cc} \Delta {\left(X_{\theta }^{2}\right)}_{ij} & =\frac{1}{2}\delta_{ij}+\sqrt{P_{i}P_{j}}\left(\cos\left({\bar{\gamma }}_{i}-{\bar{\gamma }}_{j}\right)\sinh^{2}r+\cos\left({\bar{\gamma }}_{i}+{\bar{\gamma }}_{j}\right)\sinh r\cosh r\right) \end{array}$$

(A14b) $$\begin{array}{cc} \Delta {\left(P_{\theta }^{2}\right)}_{ij} & =\frac{1}{2}\delta_{ij}+\sqrt{P_{i}P_{j}}\left(\cos\left({\bar{\gamma }}_{i}-{\bar{\gamma }}_{j}\right)\sinh^{2}r-\cos\left({\bar{\gamma }}_{i}+{\bar{\gamma }}_{j}\right)\sinh r\cosh r\right) \end{array}$$

(A14c) $$\begin{array}{cc} \Delta {\left(XP_{\theta }\right)}_{ij} & =\sqrt{P_{i}P_{j}}\left[-\sin\left({\bar{\gamma }}_{i}-{\bar{\gamma }}_{j}\right)\sinh^{2}r+\sin\left({\bar{\gamma }}_{i}+{\bar{\gamma }}_{j}\right)\sinh r\cosh r\right]. \end{array}$$

If we are performing homodyne detection only on a single channel, say the first, as it is done, for example, in Section 2, we need to first trace away the M−1 channels we are not observing. The probability density function p(x) is thus given by the marginal of the Wigner distribution of the state $\hat{U}\left(\theta \right)\hat{U}|\alpha ,r\rangle$ associated with the first symplectic variable $x_{1}$ and, equivalently, a Gaussian distribution with average μ given by the first element of $d_{\theta }$ in Equation (A12)

(A15) $$\mu =\sqrt{2}\alpha \sqrt{P_{1}}\cos{\bar{\gamma }}_{1}$$

and variance $\sigma^{2}$ given by the first row, first column element of $\Gamma_{\theta }$ in Equation (A13)

(A16) $$\sigma^{2}=\frac{1}{2}+P_{1}\left(\sinh^{2}r+\cos2{\bar{\gamma }}_{1}\sinh r\cosh r\right).$$

For a squeezed vacuum state, α=0 and we recognise the expression of the variance in Equation (6) from Equation (A16).

If, instead, we are observing all *M* output channels of the network *U*, the probability density function p(x) will be a Gaussian distribution with average μ given by the first *M* elements of $d_{\theta }$

(A17) $$\mu =\sqrt{2}\alpha \left(\begin{array}{c} \sqrt{P_{1}}\cos{\bar{\gamma }}_{1}\\ \vdots \\ \sqrt{P_{M}}\cos{\bar{\gamma }}_{M} \end{array}\right),$$

from which we recognise Equation (36), while the covariance matrix Σ is given by the first *M* rows and columns of $\Gamma_{\theta }$, i.e., by the matrix in Equation (A14a)

(A18) $$\Sigma_{ij}=\frac{1}{2}\delta_{ij}+\sqrt{P_{i}P_{j}}\left(\cos\left({\bar{\gamma }}_{i}-{\bar{\gamma }}_{j}\right)\sinh^{2}r+\cos\left({\bar{\gamma }}_{i}+{\bar{\gamma }}_{j}\right)\sinh r\cosh r\right),$$

from which we obtain the expression in Equation (35).

To evaluate the determinant |Σ| in Equation (37), we first notice that the covariance matrix Γ of the initial state |α,r〉 can be written as the sum $\Gamma \equiv I_{2M}/2+K_{0}$, where I is the 2M×2M identity matrix and $K_{0}=\Gamma -I_{2M}/2$ is a diagonal matrix of rank 2. Since the rank is invariant under orthogonal rotations, the same holds true for $\Gamma_{\theta }=R\Gamma R^{T}\equiv I_{2M}+K$, with rank(K)=2. By definition of rank, none of the sub-matrices of *K* can have rank greater than 2, hence we can write

(A19) $$\Sigma =I_{M}/2+\left(\Sigma -I_{M}/2\right)\equiv I_{M}/2+A,$$

with rank(A)≤2, since *A* is a submatrix of *K*

(A20) $$A_{ij}=\sqrt{P_{i}P_{j}}\left(\cos\left({\bar{\gamma }}_{i}-{\bar{\gamma }}_{j}\right)\sinh^{2}r+\cos\left({\bar{\gamma }}_{i}+{\bar{\gamma }}_{j}\right)\sinh r\cosh r\right)$$

We can thus apply the results in Appendix E to Σ and write $\left|\Sigma |=\right|I_{M}/2+A|$ as a sum of determinants of the matrices obtained replacing any number of columns of $I_{M}/2$, with the respective columns of *A*

(A21) $$\left|\Sigma \right|=\frac{1}{2^{M}}+\frac{1}{2^{M-1}}\sum_{i=1}^{M}A_{ii}+\frac{1}{2^{M-2}}\sum_{i=1}^{M}\sum_{j=i+1}^{M}A_{ii}A_{jj}-A_{ij}^{2},$$

where the first term is the determinant of $I_{M}/2$, the terms in the first summation are determinants of the matrices obtained by substituting the *i*-th column of $I_{M}/2$ with the *i*-th column of *A*, and the terms in the last summation from substituting the *i*-th and *j*-th columns, while also exploiting the symmetry of *A*. Noticeably, since rank A≤2, all the contributions involving the replacement of three or more columns of *A* are vanishing for the definition of rank of a matrix. Replacing the expression of *A* found in Equation (A20) into Equation (A21), the expression in Equation (37) can then be easily obtained after some elementary trigonometry.

## Appendix B. Fisher Information for Gaussian Probabilities

In this Appendix, we will evaluate the Fisher information F(φ) associated with the estimation of a parameter φ from a *M*-variate Gaussian distribution

(A22) $$p_{\varphi }\left(x\right)=\frac{1}{\sqrt{{\left(2\pi \right)}^{M}\det\left[\Sigma \right]}}\exp\left(-\frac{1}{2}{\left(x-\mu \right)}^{T}\Sigma^{-1}\left(x-\mu \right)\right),$$

where we assume that both the covariance matrix Σ and the average μ depend on the parameter φ.

Recalling the definition of the Fisher information in Equation (7), we first evaluate the logarithmic derivative

(A23) $$\begin{array}{cc} \frac{\partial }{\partial \varphi }\ln p_{\varphi }\left(x\right) & =-\frac{1}{2}\frac{\partial }{\partial \varphi }\ln\det\left[\Sigma \right]-\frac{1}{2}{\left(x-\mu \right)}^{T}\frac{\partial \Sigma^{-1}}{\partial \varphi }\left(x-\mu \right)\\ \; & \;\;\;\;+\frac{\partial \mu^{T}}{\partial \varphi }\Sigma^{-1}\left(x-\mu \right). \end{array}$$

In order to evaluate the Fisher information in Equations (9) and (38), we first notice that the expression ${\left(\frac{\partial }{\partial \varphi }\ln p_{\varphi }\left(x\right)\right)}^{2}$ contains terms of order up to fourth in (x−μ). Obtaining the Fisher information thus reduces to the evaluation of the expectation values of a polynomial in (x−μ), for which we can exploit standard results of Gaussian integrals:

(A24a) $$\begin{array}{cc} E_{p_{\varphi }}\left[x_{i}-\mu_{i}\right] & =0 \end{array}$$

(A24b) $$\begin{array}{cc} E_{p_{\varphi }}\left[\left(x_{i}-\mu_{i}\right)\left(x_{j}-\mu_{j}\right)\right] & =\Sigma_{ij} \end{array}$$

(A24c) $$\begin{array}{cc} E_{p_{\varphi }}\left[\left(x_{i}-\mu_{i}\right)\left(x_{j}-\mu_{j}\right)\left(x_{k}-\mu_{k}\right)\right] & =0 \end{array}$$

(A24d) $$\begin{array}{cc} E_{p_{\varphi }}\left[\left(x_{i}-\mu_{i}\right)\left(x_{j}-\mu_{j}\right)\left(x_{k}-\mu_{k}\right)\left(x_{l}-\mu_{l}\right)\right] & =\Sigma_{ij}\Sigma_{kl}+\Sigma_{ik}\Sigma_{jl}+\Sigma_{il}\Sigma_{jk}, \end{array}$$

from which we see that only even powers of the term (x−μ) have a non-vanishing contribution. We can thus evaluate

(A25) $$\begin{array}{cc} F(\varphi ) & =E_{p_{\varphi }}\left[{\left(\frac{\partial }{\partial \varphi }\ln p_{\varphi }\left(x\right)\right)}^{2}\right]\\ \; & =\frac{1}{4}{\left(\frac{\partial }{\partial \varphi }\ln\det\left[\Sigma \right]\right)}^{2}\\ \; & \;+\sum_{s,t=1}^{M}{\left(\frac{\partial \mu^{T}}{\partial \varphi }\Sigma^{-1}\right)}_{s}{\left(\frac{\partial \mu^{T}}{\partial \varphi }\Sigma^{-1}\right)}_{t}E_{p_{\varphi }}\left[\left(x_{s}-\mu_{s}\right)\left(x_{t}-\mu_{t}\right)\right]\\ \; & \;+\frac{1}{2}\left(\frac{\partial }{\partial \varphi }\ln\det\left[\Sigma \right]\right)\sum_{s,t=1}^{M}\frac{\partial \Sigma_{st}^{-1}}{\partial \varphi }E_{p_{\varphi }}\left[\left(x_{s}-\mu_{s}\right)\left(x_{t}-\mu_{t}\right)\right]\\ \; & \;+\frac{1}{4}\sum_{s,t,u,v=1}^{M}\frac{\partial \Sigma_{st}^{-1}}{\partial \varphi }\frac{\partial \Sigma_{uv}^{-1}}{\partial \varphi }E_{p_{\varphi }}\left[\left(x_{s}-\mu_{s}\right)\left(x_{t}-\mu_{t}\right)\left(x_{u}-\mu_{u}\right)\left(x_{v}-\mu_{v}\right)\right]. \end{array}$$

By substituting the expressions in Equations (A24) into Equation (A25), and exploiting the Jacobi’s formula for the derivative of the determinant of square matrices which assures that

(A26) $$\frac{1}{\det[\Sigma ]}\frac{\partial \det[\Sigma ]}{\partial \varphi }=\mathrm{Tr}[\Sigma^{-1}\frac{\partial \Sigma }{\partial \varphi }]\equiv -\mathrm{Tr}[\frac{\partial \Sigma^{-1}}{\partial \varphi }\Sigma ],$$

we see that most of the terms in Equation (A25) cancel out, yielding the Fisher information

(A27) $$\begin{array}{cc} F(\varphi ) & =\frac{\partial \mu^{T}}{\partial \varphi }\Sigma^{-1}\frac{\partial \mu }{\partial \varphi }+\frac{1}{2}\mathrm{Tr}[\frac{\partial \Sigma^{-1}}{\partial \varphi }\Sigma \frac{\partial \Sigma^{-1}}{\partial \varphi }\Sigma ]. \end{array}$$

We can easily reduce Equation (A27) to the Fisher information in Equation (9) for the single-homodyne case with μ=0.

To obtain the FIM in Equation (38), we first exploit the relation

(A28) $$\Sigma \frac{\partial \Sigma^{-1}}{\partial \varphi }\Sigma =-\frac{\partial \Sigma }{\partial \varphi },$$

and thus we express the covariance matrix $\Sigma^{-1}$ in terms of its cofactor matrix, i.e., $\Sigma^{-1}=C/\det\left[\Sigma \right]$, where we have exploited the symmetry of Σ so that $C^{T}=C$. We can thus rewrite, in the single-parameter case

(A29) $$\begin{array}{cc} F(\varphi ) & =\frac{\partial \mu^{T}}{\partial \varphi }\Sigma^{-1}\frac{\partial \mu }{\partial \varphi }-\frac{1}{2}\mathrm{Tr}[\frac{\partial \Sigma^{-1}}{\partial \varphi }\frac{\partial \Sigma }{\partial \varphi }]\\ \; & =\frac{\partial \mu^{T}}{\partial \varphi }\Sigma^{-1}\frac{\partial \mu }{\partial \varphi }+\frac{1}{2\det{\left[\Sigma \right]}^{2}}\frac{\partial \det[\Sigma ]}{\partial \varphi }\mathrm{Tr}[C\frac{\partial \Sigma }{\partial \varphi }]\\ \; & \;\;\;-\frac{1}{2\det[\Sigma ]}\mathrm{Tr}[\left(\frac{\partial C}{\partial \varphi }\right)\left(\frac{\partial \Sigma }{\partial \varphi }\right)] \end{array}$$

We can recognise in the second term of Equation (A29) the Jacobi’s formula for the derivative of the determinant in Equation (A26), which allows us to obtain the expression for the Fisher information shown in Equation (38)

(A30) $$\begin{array}{cc} F(\varphi ) & =\frac{\partial \mu^{T}}{\partial \varphi }\Sigma^{-1}\frac{\partial \mu }{\partial \varphi }+\frac{1}{2\det{\left[\Sigma \right]}^{2}}{\left(\frac{\partial \det[\Sigma ]}{\partial \varphi }\right)}^{2}-\frac{1}{2\det[\Sigma ]}\mathrm{Tr}[\left(\frac{\partial C}{\partial \varphi }\right)\left(\frac{\partial \Sigma }{\partial \varphi }\right)]. \end{array}$$

In order to elicit the cofactor matrix *C* in terms of the elements of Σ and thus obtain Equation (39), we first need to make some observations. First, the (s,t)-cofactor $C_{st}$ is defined as the determinant of the L−1×L−1 sub-matrix of Σ obtained by deleting its *s*-th row and *t*-th column, then multiplied by ${\left(-1\right)}^{s+t}$. This can be equivalently thought of as the determinant of the L×L matrix $\Sigma^{[s,t],1}$, where we denote with $X^{[s,t],n}$ the matrix obtained from the matrix *X* replacing all its elements in the the *s*-th row and in the *p*-th column with zeros, with the exception of the element (s,t) which is replaced by *n*, namely

(A31) $$C_{st}=\det\left[\left(\begin{array}{ccccccc} \Sigma_{11} & \ldots & \Sigma_{1t-1} & 0 & \Sigma_{1t+1} & \ldots & \Sigma_{1L}\\ \; & \vdots & \; & \vdots & \; & \vdots \\ \Sigma_{s-11} & \ldots & \Sigma_{s-1t-1} & 0 & \Sigma_{s-1t+1} & \ldots & \Sigma_{s-1L}\\ 0 & \ldots & 0 & 1 & 0 & \ldots & 0\\ \Sigma_{s+11} & \ldots & \Sigma_{s+1t-1} & 0 & \Sigma_{s+1t+1} & \ldots & \Sigma_{s+1L}\\ \; & \vdots & \; & \vdots & \; & \vdots \\ \Sigma_{L1} & \ldots & \Sigma_{Lt-1} & 0 & \Sigma_{Lt+1} & \ldots & \Sigma_{LL} \end{array}\right)\right].$$

For the particular case of the covariance matrix Σ in Equation (35), as discussed in Appendix A, we can write $\Sigma =I_{M}/2+A$, with *A* symmetric matrix such that rank(A)=ρ≤2 given in Equation (A20). Thus, we can write the (s,t)-cofactor as

(A32) $$C_{st}=\det\left[{\left(I_{M}/2\right)}^{[s,t],0}+A^{[s,t],1}\right],$$

and evaluate this determinant with the methods discussed in detail in Appendix E. In particular, $C_{st}$ is obtained as a sum of determinants of matrices obtained by replacing any possible set of columns of ${\left(I_{M}/2\right)}^{[s,t],0}$ with the columns in the same positions of $A^{[s,t],1}$. Noticeably, by substituting a row and a column of *A* with an arbitrary row or column, in general increases its rank by one, so that $\mathrm{rank}(A^{[s,t],1})\leq 3$. To evaluate the cofactor matrix *C*, it is thus convenient to consider separately the simpler case s=t first, and then s≠t.

For s=t in Equation (A32), the cofactor becomes $C_{ss}=\det\left[{\left(I_{M}/2\right)}^{[s,s],0}+A^{[s,s],1}\right]$. We notice that the matrix ${\left(I_{M}/2\right)}^{[s,s],0}$ is a diagonal matrix with a single zero eigenvalue—i.e., the *s*-th—and thus each contribution to $C_{ss}$ is non-vanishing only if the *s*-th columns of ${\left(I_{M}/2\right)}^{[s,s],0}$—a column of only 0s—is replaced. Moreover, the *s*-th column of $A^{[s,s],1}$ is also a column of all 0s, with the exception of the *s*-th element, which is equal to 1. We thus obtain

(A33) $$C_{ss}=\frac{1}{2^{M-1}}+\frac{1}{2^{M-2}}\sum_{\begin{array}{c} i=1\\ i\neq s \end{array}}^{M}A_{ii}-\frac{1}{2^{M-3}}\sum_{\begin{array}{c} i=1\\ i\neq s \end{array}}^{M}\sum_{\begin{array}{c} j=i+1\\ j\neq s \end{array}}^{M}A_{ii}A_{jj}-A_{ij}^{2},$$

which is the sum of terms obtained substituting the *s*-th, the *s*-th and *i*-th, and the *s*-th, *i*-th and *j*-th columns, respectively. Noticeably, replacing more than 3 columns yields vanishing contributions, since $\mathrm{rank}(A^{[s,t],1})\leq 3$. When s≠t, ${\left(I_{M}/2\right)}^{[s,t],0}$ is still a diagonal matrix but with two zeros in the diagonal, more precisely the *s*-th an the *t*-th elements. Both the *s*-th and the *t*-th columns are thus columns of zeros, and all non-vanishing contributions to $C_{st}$ must replace these two columns. For example, the only contribution obtained by swapping the *s*-th and *t*-th columns is of the type

(A34) $$\frac{1}{2^{M-2}}\det\left[\left(\begin{array}{cc} 0 & 1\\ A_{ts} & 0 \end{array}\right)\right]=-\frac{1}{2^{M-2}}A_{st},$$

exploiting at the same time the symmetry of *A*. The contributions obtained by swapping the *s*-th, *t*-th and *i*-th columns, with i≠s,t, are of the type

(A35) $$\frac{1}{2^{M-3}}\det\left[\left(\begin{array}{ccc} 0 & 1 & 0\\ A_{ts} & 0 & A_{ti}\\ A_{is} & 0 & A_{ii} \end{array}\right)\right]=\frac{1}{2^{M-3}}\left(A_{si}A_{ti}-A_{st}A_{ii}\right),$$

where once again, we are exploiting the symmetry of *A*. Once again, substituting more than 3 columns yields no contributions, since $\mathrm{rank}(A^{[s,t],1})\leq 3$. The final expression for $C_{st}$, s≠t thus reads

(A36) $$C_{st}=-\frac{1}{2^{M-2}}A_{st}+\frac{1}{2^{M-3}}\sum_{\begin{array}{c} i=1\\ i\neq s,t \end{array}}^{M}\left(A_{si}A_{ti}-A_{st}A_{ii}\right).$$

Replacing in (A33) and (A36) the definition of $A=\Sigma -I_{M}/2$ in Equation (A32), it is straightforward to obtain Equation (39).

## Appendix C. Asymptotic Analyses of Gaussian Metrology

In this appendix, we will perform all the asymptotic analyses for a large number of photons *N* in the setups presented in Section 2 and Section 3.

### Appendix C.1. Single Homodyne

Here, we evaluate the asymptotic expressions of the Fisher information in Equation (16), showing that the conditions in Equations (14) and (15) yield the Heisenberg scaling for the estimation of the respective quantities of interest.

As shown in Equations (10) and (11), the dependence of the variance

(A37) $$\sigma_{\varphi }^{2}=\frac{1-P_{\varphi }}{2}+\frac{P_{\varphi }}{2}\left[\cosh\left(2r\right)+\cos\left(2\gamma_{\varphi }-2\theta \right)\sinh\left(2r\right)\right],$$

on the parameters φ only appears through the transition probability $P_{\varphi }$ and the acquired complex phase $\gamma_{\varphi }$

(A38) $$\frac{\partial \sigma_{\varphi }^{2}}{\partial \varphi }=\left(\partial_{P}\sigma_{\varphi }^{2}\right)\frac{\partial P_{\varphi }}{\partial \varphi }+\left(\partial_{\gamma }\sigma_{\varphi }^{2}\right)\frac{\partial \gamma_{\varphi }}{\partial \varphi },$$

where $\partial_{P}$ and $\partial_{f}$ represent the differentiation with respect to $P_{\varphi }$ and $\gamma_{\varphi }$, and

(A39a) $$\begin{array}{cc} \partial_{P}\sigma_{\varphi }^{2} & =\frac{1}{2}\left(-1+\cosh\left(2r\right)+\cos\left(2\gamma_{\varphi }-2\theta \right)\sinh\left(2r\right)\right) \end{array}$$

(A39b) $$\begin{array}{cc} \partial_{\gamma }\sigma_{\varphi }^{2} & =-P_{\varphi }\sin\left(2\gamma_{\varphi }-2\theta \right)\sinh\left(2r\right). \end{array}$$

To achieve the Heisenberg scaling, some conditions must be imposed so that the variance in Equation (A37) does not grow with *N*. The only option to do that without ruining the sensitivity of the setup is requiring that $\gamma_{\varphi }-\theta ≃\pi /2$, as we can see from Equation (A37). In particular, we impose condition (14), and evaluate the variance in (A37) and its gradient (A38) in the large *N* limit

(A40) $$\begin{array}{cc} \sigma_{\varphi }^{2} & =\frac{1}{2}+NP_{\varphi }\left(1-\cos\left(\frac{2k}{N}\right)\sqrt{1+\frac{1}{N}}\right)\\ \; & =\frac{1}{2}+NP_{\varphi }\left(1-\left(1-\frac{2k^{2}}{N^{2}}\right)\left(1+\frac{1}{2N}-\frac{1}{8N^{2}}\right)\right)+O\left(\frac{1}{N^{2}}\right)\\ \; & =\frac{1-P_{\varphi }}{2}+P_{\varphi }\left(\frac{2k^{2}}{N}+\frac{1}{8N}\right)+O\left(\frac{1}{N^{2}}\right), \end{array}$$

(A41) $$\begin{array}{cc} \frac{\partial }{\partial \varphi }\sigma_{\varphi }^{2} & =N\frac{\partial }{\partial \varphi }P_{\varphi }\left(1-\cos\left(\frac{2k}{N}\right)\sqrt{1+\frac{1}{N}}\right)+2NP_{\varphi }\frac{\partial }{\partial \varphi }\gamma_{\varphi }\sin\left(\frac{2k}{N}\right)\sqrt{1+\frac{1}{N}}\\ \; & =N\frac{\partial }{\partial \varphi }P_{\varphi }\left(1-\left(1+\frac{1}{2N}\right)\right)+2NP_{\varphi }\frac{\partial }{\partial \varphi }\gamma_{\varphi }\frac{2k}{N}+O\left(\frac{1}{N}\right)\\ \; & =-\frac{1}{2}\frac{\partial }{\partial \varphi }P_{\varphi }+4kP_{\varphi }\frac{\partial }{\partial \varphi }\gamma_{\varphi }+O\left(\frac{1}{N}\right). \end{array}$$

Then, by imposing condition (15) on $P_{\varphi }$, we get

(A42a) $$\begin{array}{cc} \sigma_{\varphi }^{2} & =\left(2k^{2}+\frac{1}{8}+\frac{\ell }{2}\right)\frac{1}{N}+O\left(\frac{1}{N^{2}}\right), \end{array}$$

(A42b) $$\begin{array}{cc} \frac{\partial }{\partial \varphi }\sigma_{\varphi }^{2} & =4k\frac{\partial }{\partial \varphi }\gamma_{\varphi }+O\left(\frac{1}{N}\right). \end{array}$$

By substituting the expressions in Equation (A42) into the Fisher information in Equation (9), we can finally evaluate its asymptotic expression

(A43) $$F\left(\varphi \right)∼8\varrho \left(k,\ell \right)N^{2}\left(\frac{\partial \gamma_{\varphi }}{\partial \varphi }\right)\left(\frac{\partial \gamma_{\varphi }}{\partial \varphi^{T}}\right)$$

with

(A44) $$\varrho \left(k,\ell \right)={\left(\frac{8k}{16k^{2}+1+4\ell }\right)}^{2}$$

a positive and *N*-independent pre-factor.

### Appendix C.2. Multiple Homodyne

In this appendix, we will study the asymptotic regime of the Fisher information in Equation (38),

(A45) $$\begin{array}{c} F\left(\varphi \right)=\underset{F_{D}\left(\varphi \right)}{\underset{︸}{\frac{1}{\det[\Sigma ]}\partial_{\varphi }\mu^{T}C\partial_{\varphi }\mu }}+\underset{F_{S}\left(\varphi \right)}{\underset{︸}{\frac{1}{2}{\left(\frac{\partial_{\varphi }\det\left[\Sigma \right]}{\det[\Sigma ]}\right)}^{2}-\frac{1}{2\det[\Sigma ]}\mathrm{Tr}[\left(\partial_{\varphi }\Sigma \right)\left(\partial_{\varphi }C\right)]}} \end{array}$$

for large $N=N_{S}+N_{D}=\sinh^{2}r+\alpha^{2}$. We will show that conditions (41) are necessary to reach the Heisenberg scaling, and that, when they hold, the asymptotic expression for the Fisher information is the one shown in Equation (43).

As discussed in Section 3.2, all terms in the numerators of the Fisher information in Equation (A45) are at most of the order $N_{S}^{2}$ or $N_{S}N_{D}$. We thus need to study the asymptotic behaviour of the determinant det[Σ] in Equation (37), which is at the denominators of the FI, and find what conditions prevent det[Σ] from growing with $N_{S}$ as well, ruining the Heisenberg scaling. In particular, in order to reach the Heisenberg scaling, namely a scaling of the order of $N^{2}$ in the FI, the determinant det[Σ] must be at most of the order O(1).

Thus, we first focus our attention on det[Σ] in Equation (37)

(A46) $$\begin{array}{cc} \det\left[\Sigma \right]=\frac{1}{2^{M}} & +\frac{\sinh(r)}{2^{M-1}}\sum_{i=1}^{M}P_{i}\left(\sinh\left(r\right)+\cos\left(2{\bar{\gamma }}_{i}\right)\cosh\left(r\right)\right)\\ \; & -\frac{\sinh^{2}\left(r\right)}{2^{M-2}}\sum_{i=1}^{M}\sum_{j=i+1}^{M}P_{i}P_{j}\sin^{2}\left({\bar{\gamma }}_{i}-{\bar{\gamma }}_{j}\right). \end{array}$$

In particular, we will suppose that the asymptotic behaviour of the complex phases ${\bar{\gamma }}_{i}$ for large $N_{S}$ is given by ${\bar{\gamma }}_{i}={\bar{\gamma }}_{0i}+k_{i}N_{S}^{-\alpha }$, with $k_{i}\in R$ independent of *N* and α>0 (Notice that this condition is not restrictive, since if ${\bar{\gamma }}_{i}$ were to grow with *N* (i.e., α<0), it would yield an oscillating asymptotic behaviour of det[Σ] and every other term appearing in the FI). By expanding in powers of $N_{S}$ the terms in which the squeezing parameter *r* appears in (A46) we obtain

(A47) $$\begin{array}{c} \det\left[\Sigma \right]=D_{1}N_{S}+D_{2}+D_{3}\frac{1}{N_{S}}+O\left(\frac{1}{N_{S}^{2}}\right), \end{array}$$

where

(A48a) $$\begin{array}{cc} D_{1} & =\frac{1}{2^{M-1}}\left(1+\sum_{i=1}^{M}P_{i}\cos\left(2{\bar{\gamma }}_{i}\right)\right)-\frac{1}{2^{M-2}}\left(\sum_{i=1}^{M}\sum_{j=i+1}^{M}P_{i}P_{j}\sin{\left({\bar{\gamma }}_{i}-{\bar{\gamma }}_{j}\right)}^{2}\right), \end{array}$$

(A48b) $$\begin{array}{cc} D_{2} & =\frac{1}{2^{M}}\left(1+\sum_{i=1}^{M}P_{i}\cos\left(2{\bar{\gamma }}_{i}\right)\right), \end{array}$$

(A48c) $$\begin{array}{cc} D_{3} & =-\frac{1}{2^{M+2}}\sum_{i=1}^{M}P_{i}\cos\left(2{\bar{\gamma }}_{i}\right). \end{array}$$

To prevent the scaling with $N_{S}$ of the determinant det[Σ], $D_{1}$ must be set as equal to, or tending to, zero. After some trigonometry, and exploiting the fact that $\sum_{i}P_{i}=1$ due to the unitarity of the network, we can rewrite

(A49) $$D_{1}=\frac{1}{2^{M-2}}\left({\left(\sum_{i=1}^{M}P_{i}\cos^{2}{\bar{\gamma }}_{i}\right)}^{2}+{\left(\sum_{i=1}^{M}P_{i}\sin{\bar{\gamma }}_{i}\cos{\bar{\gamma }}_{i}\right)}^{2}\right),$$

which tends to zero only if both terms in the parenthesis tend to zero, i.e., when ${\bar{\gamma }}_{0i}=\pi /2+n\pi$, with n∈Z. To analyse the order for large $N_{S}$ of the determinant when the condition ${\bar{\gamma }}_{0i}=\pi /2+n\pi$ holds—and thus ${\bar{\gamma }}_{i}=\pi /2+n\pi +k_{i}N_{S}^{-\alpha }$—we need to analyse the behaviour of each term in Equation (A48). Since $\cos\left(2{\bar{\gamma }}_{i}\right)∼-1+2k_{i}N_{S}^{-2\alpha }$, and $\sin{\left({\bar{\gamma }}_{i}-{\bar{\gamma }}_{j}\right)}^{2}∼\left(k_{i}-k_{j}\right)N_{S}^{-2\alpha }$, both $D_{1}$ and $D_{2}$ scale with $N_{S}^{-2\alpha }$, while $D_{3}$ is asymptotically constant. Thus, we find the asymptotic behaviour of det[Σ] through Equation (A47): the term $D_{2}$ is always negligible with respect to $D_{1}N_{S}$, while for α≤1 the term $D_{1}N_{S}$ dominates over $D_{3}/N_{S}$ so that det[Σ] is of order $N_{S}^{1-2\alpha }$, and for α>1 instead $D_{3}/N_{S}$ dominates $D_{1}N_{S}$ and the determinant scales with $N_{S}^{-1}$. In equations:

(A50) $$\det\left[\Sigma \right]∼\left\{\begin{array}{c} D_{1}N_{S}\propto N_{S}^{1-2\alpha },\;\mathrm{for}\;\alpha \leq 1\\ D_{3}N_{S}^{-1}\propto N_{S}^{-1},\;\mathrm{for}\;\alpha >1 \end{array}\right.$$

Noticeably, the determinant stops growing with $N_{S}$ only for α≥1/2.

We thus now study the asymptotic behaviour of the numerators appearing in the Fisher information in Equation (A45), when the condition ${\bar{\gamma }}_{i}=\pi /2+k_{i}N_{S}^{-\alpha }$, with α≥1/2, is true. We will perform the same analysis done for det[Σ] earlier, considering only the dominating term for every value of α. We first obtain the derivative of Σ from Equation (35), substituting ${\bar{\gamma }}_{i}=\pi /2+k_{i}/N_{S}^{\alpha }$, with α≥1/2,

(A51) $$\begin{array}{cc} \partial_{\varphi }\Sigma_{ij}= & -\frac{1}{2}\partial_{\varphi }\left(\sqrt{P_{i}P_{j}}\right)+\sqrt{P_{i}P_{j}}(\partial_{\varphi }\left({\bar{\gamma }}_{i}+{\bar{\gamma }}_{j}\right)\left(k_{i}+k_{j}\right)\\ \; & -\partial_{\varphi }\left({\bar{\gamma }}_{i}-{\bar{\gamma }}_{j}\right)\left(k_{i}-k_{j}\right))N_{S}^{1-\alpha }+O\left(N_{S}^{1-2\alpha }\right)+O\left(N_{S}^{-1}\right), \end{array}$$

and we notice that each element of Σ scales at most with $N_{S}^{1-\alpha }$ for 1/2≤α<1, while it becomes constant for α≥1. We then analyse the auxiliary term (40)

(A52) $$\begin{array}{cc} S_{sti} & =\sinh^{2}\left(r\right)\sqrt{P_{s}P_{t}}P_{i}\sin\left({\bar{\gamma }}_{s}-{\bar{\gamma }}_{i}\right)\sin\left({\bar{\gamma }}_{t}-{\bar{\gamma }}_{i}\right)=O\left(N_{S}^{1-2\alpha }\right), \end{array}$$

of which we evaluate the derivative, always with the conditions ${\bar{\gamma }}_{i}=\pi /2+k_{i}/N_{S}^{\alpha }$, with α≥1/2

(A53) $$\begin{array}{cc} \partial_{\varphi }S_{sti} & =\sqrt{P_{s}P_{t}}P_{i}(\left(\partial_{\varphi }\left({\bar{\gamma }}_{s}-{\bar{\gamma }}_{i}\right)\right)\left(k_{t}-k_{i}\right)\\ \; & +\left(\partial_{\varphi }\left({\bar{\gamma }}_{t}-{\bar{\gamma }}_{i}\right)\right)\left(k_{s}-k_{i}\right))N_{S}^{1-\alpha }+O\left(N^{1-2\alpha }\right), \end{array}$$

which scales at most with $N_{S}^{1-\alpha }$ for large $N_{S}$. Moreover, the covariance matrix Σ in (35) asymptotically reads

(A54) $$\Sigma_{ij}=\frac{\delta_{ij}-\sqrt{P_{i}P_{j}}}{2}+O\left(N_{S}^{1-2\alpha }\right)+O\left(N_{S}^{-1}\right),$$

which is in general constant asymptotically for large $N_{S}$. Inserting Equations (A52) and (A54) in the cofactor matrix (39), we obtain

(A55) $$C_{st}=\frac{1}{2^{M-1}}\sqrt{P_{s}P_{t}}+O\left(N_{S}^{1-2\alpha }\right)+O\left(N_{S}^{-1}\right).$$

Exploiting the asymptotic behaviours of the derivatives found in Equations (A51) and (A53), we are able to analyse the remaining terms appearing in the FI

(A56) $$\begin{array}{cc} \partial_{\varphi }\det\left[\Sigma \right] & =\frac{1}{2^{M-1}}\sum_{i=1}^{M}\partial_{\varphi }\Sigma_{ii}-\frac{1}{2^{M-2}}\sum_{i=1}^{M}\sum_{j=i+1}^{M}\partial_{\varphi }S_{iij}, \end{array}$$

(A57) $$\begin{array}{cc} \partial_{\varphi }C_{ss} & =\frac{1}{2^{M-2}}\sum_{\begin{array}{c} i=1\\ i\neq s \end{array}}^{M}\partial_{\varphi }\Sigma_{ii}-\frac{1}{2^{M-3}}\sum_{\begin{array}{c} i=1\\ i\neq s \end{array}}^{M}\sum_{\begin{array}{c} j=i+1\\ j\neq s \end{array}}^{M}\partial_{\varphi }S_{iij}, \end{array}$$

(A58) $$\begin{array}{cc} \partial_{\varphi }C_{st} & =-\frac{1}{2^{M-2}}\partial_{\varphi }\Sigma_{st}+\frac{1}{2^{M-3}}\sum_{\begin{array}{c} i=1\\ i\neq s,t \end{array}}^{M}\partial_{\varphi }S_{sti}. \end{array}$$

It is easy to see that $\partial_{\varphi }\det\left[\Sigma \right]$ scales at most with $N_{S}^{1-\alpha }$, since $\sum_{i}^{M}\partial_{\varphi }P_{i}=0$, while the derivatives of the elements of *C* scale at most with $N_{S}^{1-\alpha }$ for 1/2≤α<1, and become constant for α≥1. The last step is to evaluate the asymptotic behaviour for the derivative $\partial_{\varphi }\mu$ of the average, which can be easily obtained by differentiating Equation (36)

(A59) $$\begin{array}{cc} \partial_{\varphi }\mu_{i} & =\sqrt{2N_{D}}\left(\frac{\partial_{\varphi }P_{i}}{2\sqrt{P_{i}}}\cos{\bar{\gamma }}_{i}-\sqrt{P_{i}}\partial_{\varphi }{\bar{\gamma }}_{i}\sin{\bar{\gamma }}_{i}\right)\\ \; & =-\sqrt{2N_{D}P_{i}}\partial_{\varphi }{\bar{\gamma }}_{i}+O\left(\sqrt{N_{D}}N_{S}^{-\alpha }\right). \end{array}$$

Now we can finally draw the conclusions, and obtain the scaling of the Fisher information shown in Equation (43) by putting together all the asymptotic regimes found. First, we notice that, independently of α≥1/2, the third contribution to the Fisher information in Equation (A45) always scales with $N_{S}$, hence only reaching shot-noise precision. Thus, it is always dominated by the other two terms in the Heisenberg scaling regime. The only terms which allow the Heisenberg scaling are then the first two: in fact, the term $F_{D}\left(\varphi \right)$ scales with $N_{D}N_{S}^{2\alpha -1}$ for 1/2≤α≤1, and with $N_{D}N_{S}$ for α>1, thus reaching the Heisenberg scaling for α≥1, while the second term reaches sub-shot noise scaling $N_{S}^{2-2|\alpha -1|}$ for 1/2<α<3/2, achieving the Heisenberg scaling for α=1. It is interesting to notice the major difference between the two contributions to the Heisenberg scaling: the term $F_{D}\left(\varphi \right)$ reaches the Heisenberg scaling for every value of α≥1, which means that the choice ${\bar{\gamma }}_{i}=\pi /2$—corresponding to α=+∞—still yields the Heisenberg scaling. On the other hand, α must be equal to 1 in order for the first term of $F_{S}\left(\varphi \right)$ to achieve the Heisenberg scaling. The consequences and the physical implications of this difference are discussed in depth in Section 3.2.

The conditions to reach the Heisenberg scaling can thus be condensed into the request on the complex phases

(A60) $${\bar{\gamma }}_{i}=\pi /2+O\left(N_{S}^{-1}\right),$$

for i=1,…,M, as shown in Equation (41), or equivalently that ${\bar{\gamma }}_{i}≃\pi /2+k_{i}/N_{S}$ asymptotically, with $k_{i}\in R$ independent of $N_{S}$. Moreover, we have seen that the only relevant terms under this condition are the first two in Equation (A45), and that only for α=1 the first term of $F_{S}\left(\varphi \right)$ is non-vanishing.

Finally, we can now prove (43). Substituting condition (A60) into Equations (A51) and (A53), we obtain from Equation (A56)

(A61) $$\begin{array}{cc} \partial_{\varphi }\det\left[\Sigma \right] & ∼\frac{1}{2^{M-1}}\sum_{i=1}^{M}4P_{i}k_{i}\left(\partial_{\varphi }{\bar{\gamma }}_{i}\right)-\frac{1}{2^{M-2}}\sum_{i=1}^{M}\sum_{j=i+1}^{M}2P_{i}P_{j}\left(k_{i}-k_{j}\right)\left(\partial_{\varphi }{\bar{\gamma }}_{i}-\partial_{\varphi }{\bar{\gamma }}_{j}\right)\\ \; & ∼\frac{1}{2^{M-3}}\sum_{i=1}^{M}P_{i}k_{i}\sum_{j=1}^{M}P_{j}\partial_{\varphi }{\bar{\gamma }}_{j} \end{array}$$

where we exploited the passivity of the interferometer which assures $\sum_{i=1}^{M}P_{i}=1$. We also obtain from (A46)

(A62) $$\det\left[\Sigma \right]≃\frac{1}{2^{M-2}N_{S}}\left({\left(\sum_{i=1}^{M}P_{i}k_{i}\right)}^{2}+\frac{1}{16}\right),$$

which coincides with the expression in Equation (42). Lastly, from Equation (A59) when conditions (A60) hold, we obtain the asymptotic behaviour

(A63) $$\partial_{\varphi }\mu_{i}∼-\sqrt{2N_{D}P_{i}}\partial_{\varphi }{\bar{\gamma }}_{i}.$$

Substituting Equations (A55) and (A61)–(A63) into the Fisher information in Equation (38) yields the asymptotic expression shown in Equation (43).

## Appendix D. Maximum-Likelihood Estimators for Gaussian Distributions

In this Appendix, we will derive the Likelihood equation associated with the estimation schemes presented in Section 2 and Section 3, i.e., the equation whose solution maximises the Likelihood functions in Equations (18) and (46). In doing so, we will demonstrate the expressions in Equations (19) and (47).

We first suppose that the *M*-variate Gaussian probability distribution

(A64) $$p_{\varphi }\left(x\right)=\frac{1}{\sqrt{{\left(2\pi \right)}^{M}\det\left[\Sigma \right]}}\exp\left(-{\left(x-\mu \right)}^{T}\Sigma^{-1}\left(x-\mu \right)\right)$$

governing the outcomes $x=(x_{1},\ldots ,x_{M})$ depends on a single parameter φ through its average μ and its covariance matrix Σ. If we perform the observation ν times $x_{1},\ldots ,x_{\nu }$, the Likelihood function becomes

(A65) $$L\left(\varphi ;x_{1},\ldots ,x_{\nu }\right)=\frac{1}{\sqrt{{\left(2\pi \right)}^{M\nu }\det{\left[\Sigma \right]}^{\nu }}}\exp\left(-\frac{1}{2}\sum_{i=1}^{\nu }{\left(x_{i}-\mu \right)}^{T}\Sigma^{-1}\left(x_{i}-\mu \right)\right).$$

The maximum-likelihood estimator is obtained maximising the Likelihood function in Equation (A65)—or its logarithm. Applying the maximisation to the likelihood function in Equation (A65), we obtain the equation

(A66) $$\begin{array}{cc} 0 & =\partial_{\varphi }\ln L\left(\varphi ;x_{1},\ldots ,x_{\nu }\right){|}_{\varphi ={\tilde{\varphi }}_{\mathrm{MLE}}}\\ \; & =\partial_{\varphi }\sum_{j=1}^{\nu }\ln p\left(x_{j};\varphi \right){|}_{\varphi ={\tilde{\varphi }}_{\mathrm{MLE}}}\\ \; & ={\left[-\frac{\nu }{2}\partial_{\varphi }\ln\left(\det\left[\Sigma \right]\right)-\frac{1}{2}\partial_{\varphi }\sum_{j=1}^{\nu }{\left(x_{j}-\mu \right)}^{T}\Sigma^{-1}\left(x_{j}-\mu \right)\right]}_{\varphi ={\tilde{\varphi }}_{\mathrm{MLE}}}\\ \; & ={\left[-\frac{\nu }{2}\mathrm{Tr}[\Sigma^{-1}\partial_{\varphi }\Sigma ]-\frac{1}{2}\partial_{\varphi }\sum_{j=1}^{\nu }\mathrm{Tr}[\Sigma^{-1}\left(x_{j}-\mu \right){\left(x_{j}-\mu \right)}^{T}]\right]}_{\varphi ={\tilde{\varphi }}_{\mathrm{MLE}}}\\ \; & =\frac{1}{2}\mathrm{Tr}{\left[\partial_{\varphi }\Sigma^{-1}\left(\nu \Sigma -\sum_{j=1}^{\nu }\left(x_{j}-\mu \right){\left(x_{j}-\mu \right)}^{T}\right)\right]}_{\varphi ={\tilde{\varphi }}_{\mathrm{MLE}}}\\ \; & \;+{\left[{\left(\partial_{\varphi }\mu \right)}^{T}\Sigma^{-1}\left(\nu \mu -\sum_{j=1}^{\nu }x_{j}\right)\right]}_{\varphi ={\tilde{\varphi }}_{\mathrm{MLE}}}, \end{array}$$

where we exploited Jacobi’s formula for the derivative of the determinant of a matrix shown in Equation (A26), the identity in Equation (A28), and the symmetry of Σ. Equation (A66) is the expression of the Likelihood Equation (47). This can be easily simplified to the case of univariate Gaussian distribution for M=1 and absence of average μ, for which $\Sigma \to \sigma^{2}$ and $x_{j}\to x_{j}$, obtaining

(A67) $$0=\Sigma -\frac{1}{\nu }\sum_{j=1}^{\nu }x_{j}^{2}$$

as shown in Equation (19).

## Appendix E. Formulas for the Determinant of a Sum of Two Matrices

Let us consider an L×L matrix *Z* of the form Z=D+W, where $D=\mathrm{diag}(d_{1},\ldots ,d_{L})$ is a diagonal matrix, and rank(W)=ρ≤L. In this appendix, we will show a convenient method to express the determinant det[Z] in terms of the elements of *W*, for the evaluation of the determinant in Equation (37) and of the cofactor matrix in Equations (39).

We exploit the identity [47]

(A68) $$\det\left[Z\right]=\det\left[D+W\right]=\sum_{\alpha =0}^{L}\Theta_{\alpha }\left(D,W\right),$$

where $\Theta_{\alpha }\left(X,Y\right)$ is the sum of the determinants of the matrices obtained by replacing any set of α columns (rows) of *X* with the α columns (rows) of *Y* at the same position. For example, for two 3×3 matrices

(A69) $$X=\left(\begin{array}{ccc} X_{11} & X_{12} & X_{13}\\ X_{21} & X_{22} & X_{23}\\ X_{31} & X_{32} & X_{33} \end{array}\right)\;Y=\left(\begin{array}{ccc} Y_{11} & Y_{12} & Y_{13}\\ Y_{21} & Y_{22} & Y_{23}\\ Y_{31} & Y_{32} & Y_{33} \end{array}\right),$$

the quantity $\Theta_{\alpha }\left(D,W\right)$ is given, for α=1, by

(A70) $$\Theta_{1}\left(X,Y\right)=\det\left[\left(\begin{array}{ccc} Y_{11} & X_{12} & X_{13}\\ Y_{21} & X_{22} & X_{23}\\ Y_{31} & X_{32} & X_{33} \end{array}\right)\right]+\det\left[\left(\begin{array}{ccc} X_{11} & Y_{12} & X_{13}\\ X_{21} & Y_{22} & X_{23}\\ X_{31} & Y_{32} & X_{33} \end{array}\right)\right]+\det\left[\left(\begin{array}{ccc} X_{11} & X_{12} & Y_{13}\\ X_{21} & X_{22} & Y_{23}\\ X_{31} & X_{32} & Y_{33} \end{array}\right)\right],$$

while for α=2

(A71) $$\Theta_{2}\left(X,Y\right)=\det\left[\left(\begin{array}{ccc} Y_{11} & Y_{12} & X_{13}\\ Y_{21} & Y_{22} & X_{23}\\ Y_{31} & Y_{32} & X_{33} \end{array}\right)\right]+\det\left[\left(\begin{array}{ccc} X_{11} & Y_{12} & Y_{13}\\ X_{21} & Y_{22} & Y_{23}\\ X_{31} & Y_{32} & Y_{33} \end{array}\right)\right]+\det\left[\left(\begin{array}{ccc} Y_{11} & X_{12} & Y_{13}\\ Y_{21} & X_{22} & Y_{23}\\ Y_{31} & X_{32} & Y_{33} \end{array}\right)\right].$$

Since rank(W)=ρ, the determinant of any α×α sub-matrix of *W* is zero if α>ρ, so we can write

(A72) $$\det\left[Z\right]=\sum_{\alpha =0}^{\rho }\Theta_{\alpha }\left(D,W\right).$$

Let us now make explicit the first, easier terms of the summation. In particular, with the goal of applying this expression to the covariance matrix Σ in Equation (35) and to the cofactor matrix *C* in Equation (39), which can both be expressed as the sum of a diagonal matrix and a rank-two and rank-three matrix (see Equations (A19), (A20) and (A32)), we will explicitly report the first three terms $\Theta_{0}\left(X,Y\right)$, $\Theta_{1}\left(X,Y\right)$ and $\Theta_{2}\left(X,Y\right)$.

For α=0, no columns are replaced from *D*, hence $\Theta_{0}\left(D,W\right)=\det\left[D\right]=\prod_{k}d_{k}$. We also notice that, if at least one of the eigenvalues of *D* is zero, this term vanishes.

For α=1, $\Theta_{1}\left(D,W\right)$ is the sum of determinants of matrices of the form

(A73) $$\left(\begin{array}{ccccccccc} d_{1} & 0 & \ldots & 0 & W_{1i} & 0 & \ldots & 0 & 0\\ 0 & d_{2} & \ldots & 0 & W_{2i} & 0 & \ldots & 0 & 0\\ \; & \; & \ddots & \; & \vdots & \; & \vdots & \\ 0 & 0 & \ldots & d_{i-1} & W_{i-1i} & 0 & \ldots & 0 & 0\\ 0 & 0 & \ldots & 0 & W_{ii} & 0 & \ldots & 0 & 0\\ 0 & 0 & \ldots & 0 & W_{i+1i} & d_{i+1} & \ldots & 0 & 0\\ \; & \; & \vdots & \; & \vdots & \; & \ddots & \\ 0 & 0 & \ldots & 0 & W_{L-1i} & 0 & \ldots & d_{L-1} & 0\\ 0 & 0 & \ldots & 0 & W_{Li} & 0 & \ldots & 0 & d_{L} \end{array}\right)$$

with i=1,…,L. The determinant of a matrix of the type in Equation (A73) is straightforward, and reduces to $W_{ii}\times \prod_{k\neq i}d_{k}$. Thus, in general, $\Theta_{1}\left(D,W\right)=\sum_{i}W_{ii}\times \prod_{k\neq i}d_{k}$. A key observation to make, in view of the application to the covariance matrix Σ and cofactor matrix *C*, is that if two or more eigenvalues of *D* are zero, all these determinants are zero, and thus $\Theta_{1}\left(D,W\right)=0$; if, instead, a single eigenvalue is zero, say $d_{j}=0$, only one of these determinants of $\Theta_{1}\left(D,W\right)$ is non-vanishing, namely the determinant of the matrix obtained by replacing exactly the *j*-th column of *D*. In this case, for $d_{j}=0$, then we have $\Theta_{1}\left(D,W\right)=W_{jj}\times \prod_{k\neq j}d_{k}$.

Let us now consider lastly the case α=2. The matrices contributing to $\Theta_{2}\left(D,W\right)$ are of the form

(A74) $$\left(\begin{array}{ccccccccccc} d_{1} & \ldots & 0 & W_{1i} & 0 & \ldots & 0 & W_{1i^{\prime }} & 0 & \ldots & 0\\ \; & \ddots & \; & \vdots & \; & \vdots & \; & \vdots & \; & \vdots & \\ 0 & \ldots & d_{i-1} & W_{i-1i} & 0 & \ldots & 0 & W_{i-1i^{\prime }} & 0 & \ldots & 0\\ 0 & \ldots & 0 & W_{ii} & 0 & \ldots & 0 & W_{ii^{\prime }} & 0 & \ldots & 0\\ 0 & \ldots & 0 & W_{i+1i} & d_{i+1} & \ldots & 0 & W_{i+1i^{\prime }} & 0 & \ldots & 0\\ \; & \vdots & \; & \vdots & \; & \ddots & \; & \vdots & \; & \vdots & \\ 0 & \ldots & 0 & W_{i^{\prime }-1i} & 0 & \ldots & d_{i^{\prime }-1} & W_{i^{\prime }-1i^{\prime }} & 0 & \ldots & 0\\ 0 & \ldots & 0 & W_{i^{\prime }i} & 0 & \ldots & 0 & W_{i^{\prime }i^{\prime }} & 0 & \ldots & 0\\ 0 & \ldots & 0 & W_{i^{\prime }+1i} & 0 & \ldots & 0 & W_{i^{\prime }+1i^{\prime }} & d_{i^{\prime }+1} & \ldots & 0\\ \; & \vdots & \; & \vdots & \; & \vdots & \; & \vdots & \; & \ddots & \\ 0 & \ldots & 0 & W_{Li} & 0 & \ldots & 0 & W_{Li^{\prime }} & 0 & \ldots & d_{L} \end{array}\right),$$

with $i<i^{\prime }=1,\ldots ,L$. Once again, the determinants of the matrices of the type in Equation (A74) are easy to be evaluated, and read $\det\left[W^{\left(i,i^{\prime }\right)}\right]\;\prod_{k\notin \{i,i^{\prime }\}}d_{k}$, where

(A75) $$W^{\left(i,i^{\prime }\right)}=\left(\begin{array}{cc} W_{ii} & W_{ii^{\prime }}\\ W_{i^{\prime }i} & W_{i^{\prime }i^{\prime }} \end{array}\right).$$

We notice that $\det\left[W^{\left(i,i^{\prime }\right)}\right]=\det\left[W^{\left(i^{\prime },i\right)}\right]$. Thus, we can rewrite the terms $\Theta_{2}\left(D,W\right)$ as the sum $\Theta_{2}\left(D,W\right)=\sum_{i}\sum_{i^{\prime }>1}\det\left[W^{\left(i,i^{\prime }\right)}\right]\;\prod_{k\notin \{i,i^{\prime }\}}d_{k}$. Once again, key observations can be made: if the diagonal matrix *D* has at least three null eigenvalues, then $\Theta_{2}\left(D,W\right)$ is vanishing. If only two eigenvalues are zero, e.g., $d_{j}=d_{j^{\prime }}=0$, with $j\neq j^{\prime }$, then there is only one non-vanishing contribution to $\Theta_{2}\left(D,W\right)$, given by the matrix obtained by substituting the *j*-th and $j^{\prime }$-th columns of *D*, and in this case we have $\Theta_{2}\left(D,W\right)=\det\left[W^{\left(j,j^{\prime }\right)}\right]\;\prod_{k\notin \{j,j^{\prime }\}}d_{k}$. If only one eigenvalue is zero, namely $d_{j}=0$, then $\Theta_{2}\left(D,W\right)$ is given by the sum of all the determinants of the matrices where the *j*-th column of *D* has been replaced, namely $\Theta_{2}\left(D,W\right)=\sum_{i\neq j}\det\left[W^{\left(i,j\right)}\right]\;\prod_{k\neq i,j}d_{k}$.

It is then possible to extend with similar considerations the same method for every value of α, eventually obtaining the compact expression for the determinant in Equation (A68)

(A76) $$\det\left[Z\right]=\sum_{\alpha =0}^{\rho }\sum_{\gamma \in C_{\alpha }^{L}}\det\left[W^{\left(\gamma \right)}\right]\;\prod_{k\notin \gamma }d_{k},$$

where $C_{\alpha }^{L}$ is the set of all the combinations without repetitions of α columns out of *L*, $W^{\left(\gamma \right)}$ denotes the α×α sub-matrix of *W* obtained by selecting the rows and columns with indices $\gamma_{1},\ldots ,\gamma_{\alpha }$.
